# Roll-to-roll gravure-printed flexible perovskite solar cells using eco-friendly antisolvent bathing with wide processing window

**DOI:** 10.1038/s41467-020-18940-5

**Published:** 2020-10-13

**Authors:** Young Yun Kim, Tae-Youl Yang, Riikka Suhonen, Antti Kemppainen, Kyeongil Hwang, Nam Joong Jeon, Jangwon Seo

**Affiliations:** 1 Division of Advanced Materials, Korea Research Institute of Chemical Technology (KRICT), 141 Gajeong-ro, Yuseong-gu, Daejeon 34114 Republic of Korea; 2 Department of Materials Science and Engineering, Chungnam National University, 99 Daehak-ro, Yuseong-gu, Daejeon 34134 Republic of Korea; 3 Printed electronics processing, VTT Technical Research Centre of Finland Ltd, Kaitoväylä 1, Oulu, 90571 Finland

**Keywords:** Energy, Solar cells, Solar cells

## Abstract

Driven by recent improvements in efficiency and stability of perovskite solar cells (PSCs), upscaling of PSCs has come to be regarded as the next step. Specifically, a high-throughput, low-cost roll-to-roll (R2R) processes would be a breakthrough to realize the commercialization of PSCs, with uniform formation of precursor wet film and complete conversion to perovskite phase via R2R-compatible processes necessary to accomplish this goal. Herein, we demonstrate the pilot-scale, fully R2R manufacturing of all the layers except for electrodes in PSCs. Tert-butyl alcohol (tBuOH) is introduced as an eco-friendly antisolvent with a wide processing window. Highly crystalline, uniform formamidinium (FA)-based perovskite formation via tBuOH:EA bathing was confirmed by achieving high power conversion efficiencies (PCEs) of 23.5% for glass-based spin-coated PSCs, and 19.1% for gravure-printed flexible PSCs. As an extended work, R2R gravure-printing and tBuOH:EA bathing resulted in the highest PCE reported for R2R-processed PSCs, 16.7% for PSCs with R2R-processed SnO_2_/FA-perovskite, and 13.8% for fully R2R-produced PSCs.

## Abstract (web summary)

Driven by recent improvement in efficiency and stability of perovskite solar cells, the next step toward commercialisation is upscaling. Here, the authors demonstrate pilot-scale fully roll-to-roll manufacturing of flexible perovskite solar cells through gravure-printing and antisolvent bathing.

## Introduction

Perovskite solar cells (PSCs) have been intensively investigated as emerging photovoltaics (PVs) owing to the superior inherent advantages of perovskite as a photo-absorber such as long carrier diffusion length, high defect tolerance, high carrier mobility and high absorption coefficient^1–6^. Another crucial benefit of perovskite material is a feasible solution process to deposit thin film, which allows a cost-effective production of highly efficient PVs^7,8^. So far, both efficiency and stability of PSCs have been considerably improved, but the scalable fabrication remains a big challenge for the successful commercialization of PSCs. Specifically, the roll-to-roll (R2R) process enables high-throughput, low-cost production of flexible and light-weight PSCs, which will expand the applicability of PSCs into a variety of mobile electronic devices, vehicle- and building-integrated PVs^9,10^. There have been only a few reports on the R2R production of PSCs so far, and even they are only about partial R2R production or R2R running at laboratory scale^11–16^.

**Fig. 1:**
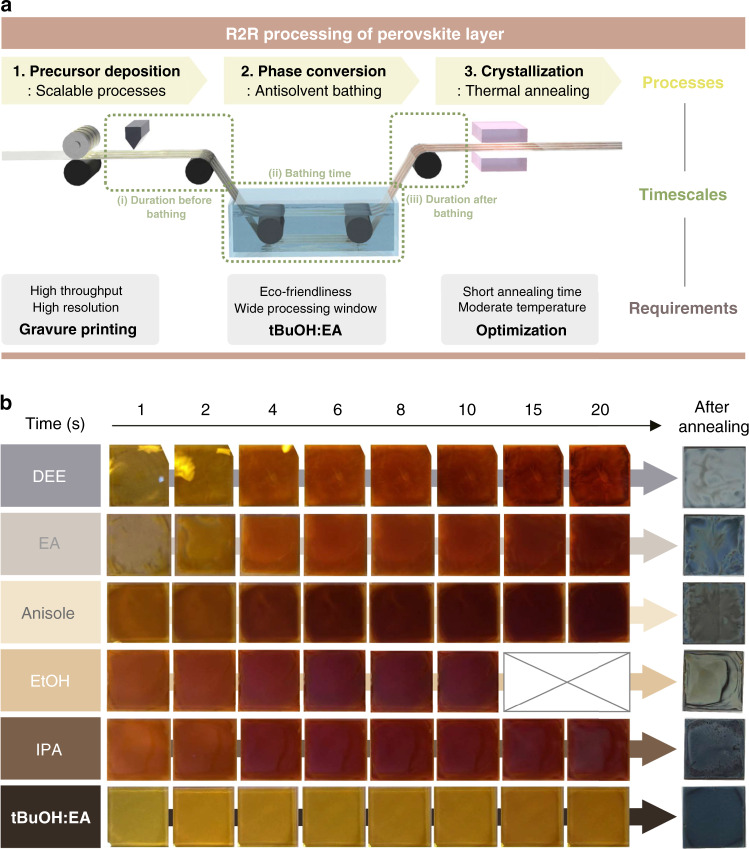
Formation of high-quality perovskite layers via antisolvent bathing. **a** Schematic diagram representing R2R processing of perovskite layer. Each step of processing, considerations of each step, and timescales are presented in the figure. **b** Photographs of perovskite film after bathing in various antisolvents. The images were recorded as a video and captured at certain times. Final films were obtained by thermal annealing of the intermediate film after bathing for 20 s.

In order to realize R2R production of PSCs, uniform, large-area deposition of sequential layers should be achieved. The R2R deposition of charge-transporting layers was already established by many researchers in organic PVs, but R2R deposition of perovskite layers has not yet been fully explored^17,18^. The R2R deposition of perovskite layers contain three key steps; (1) deposition of lead halide precursor through scalable coating techniques, (2) conversion of wet film of precursor state into supersaturation state through solvent removal, and (3) crystallization through thermal annealing. (Fig. 1a).

Uniform, thickness-controlled deposition of precursor solution is important because the uniformity and morphology of the precursor wet-film determine the quality of the resulting perovskite film grown by heterogeneous nucleation and growth. Among various scalable coating methods, gravure printing enables a simultaneous deposition and patterning of a desired layer with high resolution at relatively fast speed^15,18,19^. The potential of gravure printing for PSCs has been proved by us, successfully demonstrating a fabrication of methylammonium lead iodide (MAPbI_3_) PSCs^15^.

The conversion of wet film of precursor state into the intermediate phase has been considered as a critical step to realizing large-area, scalable production of perovskite film. The solvent-engineering method has been widely utilized to achieve high power conversion efficiencies (PCE) in a small-area device of PSCs by reaching a supersaturation state via a rapid antisolvent dripping during the spin-coating^5^. In general, the dripping process involves the physical wiping of solvents driven by centrifugal forces and solvent extraction by chemical affinity. Therefore, this led to a formation of the supersaturated film from the precursor wet film with the retardation mediator by washing out the excess solvent upon the spin-coating.

The dripping process, however, should be modified to be well-fitted into R2R deposition process of large-area perovskite layers. Various alternative methods have been reported including gas-blowing^20^, vacuum flash^21^, hot-substrate deposition^16,22,23^, and flash infrared annealing (FIRA)^24^, however, they have still several problems to be solved for R2R applications, which are the use of expensive apparatus, questionable reproducibility, or narrow processing window.

In this work, we choose the antisolvent-bathing approach for R2R fabrication. The bathing method is the most analogous method to the dripping with a view of the extraction of excess solvent from precursor wet film^25,26^. However, the bathing method requires more precise control of processing conditions to reach a high level of supersaturation state for inducing rapid crystallization. In particular, formamidinium lead iodide (FAPbI_3_) has more complex crystallization behavior with narrow processing window, which makes it more difficult to achieve the high-quality layer via bathing. The bathing method also demands a large amount of solvents, and inevitably discharges the waste solvent. Therefore, antisolvent should meet some criteria such as eco-friendliness and controllable processing window for a practical R2R production at pilot-scale. Several attempts have been made to utilize eco-friendly antisolvents in PSCs, but their usage has been limited only to dripping^27,28^. In addition, the processing windows of antisolvents have not been yet considered thoroughly, especially for bathing^29^.

As a final step, the thermal annealing is crucial for inducing a highly crystalline perovskite film upon crystal nucleation/growth step. The temperature must be lower than the processing temperature limit of the polymer substrate. Shorter annealing time is also preferred to achieve high throughput.

Herein, we demonstrate R2R-processed flexible PSCs at pilot scale through gravure printing, antisolvent bathing and subsequent annealing process. Tert-butanol (tBuOH) is introduced as an eco-friendly solvent into antisolvent-bathing, thereby generating a highly crystalline and uniform perovskite film with wide processing window. As a result of bathing in tBuOH:EA, we achieve a champion PCE of 23.5% (in reverse scan) from the glass-used device prepared by spin-coating. The gravure printing of FAPbI_3_-based perovskite layers is conducted with aid of wide processing window of tBuOH. The best PCE of 19.1% is achieved in gravure-printed flexible PSCs, which is comparable to the highest PCE of spin-coated flexible PSCs. Full R2R gravure printing of flexible PSCs except for top electrodes is demonstrated at pilot-scale via process optimization to produce a 100-m-long roll. The PSCs fabricated by R2R printed SnO_2_/perovskite exhibit a PCE of 16.7% and 16.5% when N2,N2,N2′,N2′,N7,N7,N7′,N7′-octakis(4-methoxyphenyl)-9,9′-spirobi[9H-fluorene]-2,2′,7,7′-tetramine (Spiro-OMeTAD) is spin-coated and shear-coated as HTL, respectively. The fully R2R printed PSCs with poly(3-hexylthiophene) (P3HT) as HTL show the best PCE of 13.8%, which is the highest PCE ever reported for a fully R2R-processed PSCs.

## Results

### Comparison of various eco-friendly antisolvents

In this work, tBuOH is newly suggested as an eco-friendly antisolvent. Various antisolvents are compared under various safety criteria in Supplementary Table 1^30–32^. Common antisolvents widely used for fabricating high-quality perovskite layers are as follows; diethyl ether (DEE), chlorobenzene (CB), chloroform (CF), and toluene (Tol). However, all of them are harmful to human health. We selected eco-friendly alternative antisolvents, such as alcohols or esters, or anisole. Specifically, ethanol (EtOH) and 2-propanol (IPA) as normal-alkyl alcohols, ethyl acetate (EA) as an ester, and anisole as an aromatic were chosen. We also suggested an antisolvent, tBuOH, a tert-alkyl alcohol, which is eco-friendly and has a polarity between those of normal-alkyl alcohols and esters, thereby being expected to show different behavior during antisolvent bathing. Since tBuOH has a melting point of around 30 °C, we added a small amount of EA to ensure the liquid state of the antisolvent at room temperature. DEE was also selected as a reference.

The time evolutions in appearance and morphology of as-prepared films of FAPbI_3_ after bathing using various antisolvents were investigated (Fig. 1b). As can be seen in Fig. 1b, prolonged bathing in the antisolvents except for tBuOH:EA gives rise to a dark wine-red appearance to the film after only a few seconds. This indicates that a nucleation/growth for the generation of perovskite phase occurs at the same time during the antisolvent-bathing process at room temperature. In addition, specific irregular stains with inhomogeneity are found on the film surface. Such characteristic pattern on the intermediate film is directly transferred to the final film, and the darker intermediate film has a hazy appearance after annealing. The bathing time in these antisolvents should be very short at about 1–2 s, so as to reduce haziness on the surface, and the films do not seem uniform even under that condition. In the case of tBuOH:EA, however, intermediate films look uniform in appearance and only become slightly darker as time goes on. The final perovskite film obtained by bathing in tBuOH:EA looks uniform and consistent appearance irrespective of the bathing time.

**Fig. 2:**
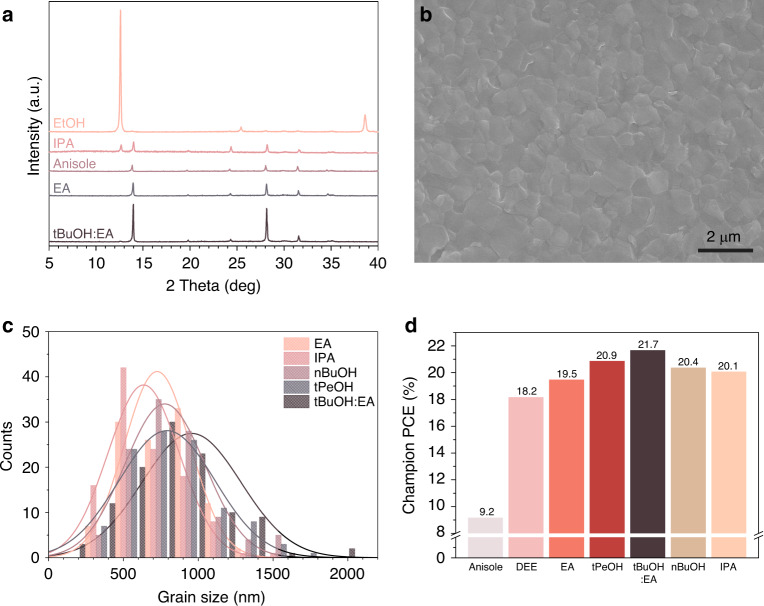
Characterization of perovskite layers formed by bathing in various antisolvent. **a** XRD spectra of perovskite films obtained by bathing in various antisolvents. **b** SEM image of the perovskite film made by bathing in tBuOH:EA. **c** Grain size distribution curves for perovskite films fabricated with various antisolvents. **d** Champion PCE of PSCs fabricated by bathing in various antisolvents.

We attempted to reveal the origins of the different behaviors upon bathing by directly mixing a precursor solution with the antisolvents (Supplementary Fig. 1, and Supplementary Note 1). As not expected for the bathing method, it was found that DEE is not effective in extracting DMF and DMSO. EA and anisole can extract the solvents, but they also disperse some of the FAPbI_3_, leading to inhomogeneity in the intermediate film with irregular stains and haziness after bathing. It is obvious that IPA partially dissolves FAI while extracting PbI_2_-DMSO from FAPbI_3_ solution (See Fig. 2a). It is worth noting that tBuOH:EA can extract DMF and excess DMSO without dispersing FAPbI_3_ or dissolving FAI in the solution. This aspect explains the homogeneous, uniform FAPbI_3_ films made using tBuOH:EA. The strong chemical interaction of tBuOH:EA with DMF and DMSO was also confirmed by the shift of the Fourier-transform Infrared (FT-IR) spectra (Supplementary Fig. 2).

FAPbI_3_ film fabricated by using tBuOH:EA as antisolvent looks uniform and highly crystalline with no trace of PbI_2_ (Fig. 2a). FAPbI_3_ films made using other alcohols have distinct peak at around 12.5°, indicating the generation of PbI_2_. It was probably due to the partial dissolution of FAI. FAPbI_3_ prepared by using DEE, EA, anisole, and tBuOH:EA showed no peaks of PbI_2_; in particular, for tBuOH:EA, the film had the highest crystallinity among all the samples.

To gain into more insight of the alkyl effect of alcohol derivatives as antisolvent, we selected n-butyl alcohol (nBuOH) and t-pentyl alcohol (tPeOH) for comparison with tBuOH. nBuOH has similar viscosity to tBuOH while tPeOH has similar polarity to tBuOH^33^ (Supplementary Figs. 3 and 4, and Supplementary Table 1). The prepared film using nBuOH looks similar to that of other normal-alkyl alcohols. The irregular stains are observed on the surface and a trace of PbI_2_ is also found from XRD pattern^34^. In contrast, the quality and the appearance of the film fabricated using tPeOH looks entirely consistent with that using tBuOH:EA. The role of the antisolvent is to extract the retardation mediator and the excess processing solvent without dissolving the perovskite precursors from the wet film. It is elucidated from this result that the polarity of the antisolvent should be considered to ensure a good quality film with uniform and consistent morphology even for a long bathing time.

The fabricated film using tBuOH:EA has a smooth morphology with a large grain size of ~1 μm (Fig. 2b). In the case of EtOH and anisole, the films have a poor surface coverage with a relatively rough surface (Supplementary Fig. 5). In contrast, in the case of tPeOH, nBuOH, EA, and IPA, the films exhibit a full surface coverage without the pinholes. The sizes of grains were measured using ImageJ software; results were averaged over 110 grains^35^ (Fig. 2c). The average grain size of FAPbI_3_ fabricated using tBuOH:EA is 955 ± 330 nm, which is much larger than that of FAPbI_3_ obtained from the other solvents. (791 ± 319, 777 ± 267, 726 ± 221, and 636 ± 238 nm from tPeOH, nBuOH, EA, and IPA, respectively).

We have investigated fluorescent lifetime images (FLIM) and their corresponding time-resolved photoluminescent (TRPL) spectra (Supplementary Fig. 6). From FLIM images, the distribution of the PL lifetimes in the entire area for the film processed with tBuOH:EA is much more even as compared to that with DEE. Its average PL lifetime is found to be 101 ns, which is higher than those of the films processed with DEE and EA (60 and 53 ns, respectively). This can support a formation of a highly crystalline and defect-less perovskite film with a good uniformity after the treatment with tBuOH:EA. The champion PCE of tBuOH:EA-used PSCs is 21.7%, which is the highest among PSCs treated with various antisolvents (Fig. 2d). The photovoltaic parameters of the champion PCE of PSCs treated with various antisolvents are summarized in Supplementary Table 2. The PCE values of PSCs processed with other antisolvents except for DEE and anisole are around 20%. In particular, a low PCE of 9.2% for the device fabricated using anisole is mainly attributed to a poor morphology of the perovskite film (as shown in Supplementary Fig. 5).

### Processing window of new antisolvent and device performances

As expected from the bathing results of Fig. 2b, a wide processing-time window could be anticipated when tBuOH:EA is used for the antisolvent bathing. To verify this, we design the modified device fabrication by mimicking R2R process of a perovskite film as follows; sequential procedures of spin-coating of precursor, stopping at a desired time, subsequent antisolvent bathing, air blowing, and annealing.

Firstly, the processing tolerance for the amount of DMSO was investigated. The amount of DMSO in the precursor solution is a critical factor determining the amount of solvents left in the precursor wet film after deposition, because DMSO has a high boiling point and strong affinity to perovskite precursors. Adjusting the amount of DMSO can alter the maximum duration before unwanted and rapid phase conversion begins, thereby allowing optimal control of the duration between printing and bathing^36^. The use of higher amount of DMSO than 1 equivalent to FAPbI_3_ in the perovskite precursor solution affords uneven and hazy perovskite films when other common antisolvents, e.g., EA and DEE are used (Supplementary Fig. 7). In the case of tBuOH:EA, however, the perovskite film looks very uniform without any haziness regardless of the amount of DMSO, from 0.7 to 2 equivalent molar amount to FAPbI_3_. This tendency is also confirmed in the scanning electron microscope (SEM) images (Supplementary Fig. 8). The perovskite films made by tBuOH:EA looked uniform and smooth regardless of the amount of DMSO, while the film made by EA or DEE with an amount of DMSO >1 showed a very rough and non-uniform morphology.

**Fig. 3:**
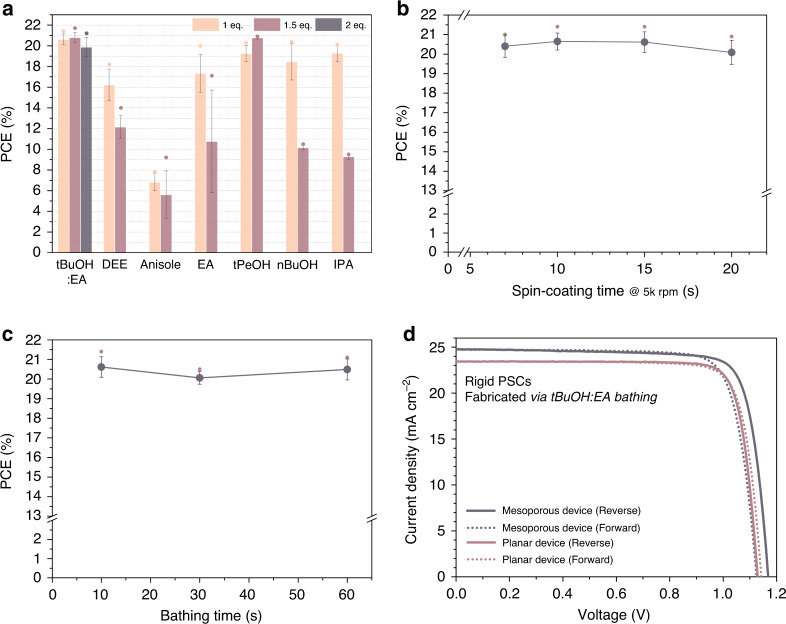
Processing window of perovskite formation processes via antisolvent bathing and champion device performance. **a** Average (bar) and champion PCE (circle) of PSCs made by bathing in antisolvents according to amounts of DMSO in the precursor solution with respect to the amount of perovskite. The gray error bars indicate standard deviation of PCEs. **b** PCE of tBuOH:EA derived PSCs depending on spin-coating duration. **c** PCE of PSCs fabricated by tBuOH:EA bathing for controlled time. In both figures, indigo dots represent average PCEs, bars indicate standard deviations, and small purple dots mean champion PCEs. **d** Current density-voltage (J-V) curves of champion devices fabricated by bathing in tBuOH:EA. Average PCE values were obtained from at least 12 devices for each condition.

Accordingly, PSCs processed by tBuOH:EA maintain their PCE values with an increasing amount of DMSO (Fig. 3a). PSCs made using other antisolvents exhibit a huge drop of PCE with increasing amount of DMSO except for tPeOH which has a tendency identical to that of tBuOH. The amount of solvent left in the wet film can be also controlled by adjusting the spin-coating duration (Fig. 3b). As expected, a longer spin-coating time can result in a larger amount of the solvent evaporation during the spin-coating. For the devices using tBuOH:EA, high average efficiencies above 20% are found irrespective of the spin-coating duration. Furthermore, it is noted that varying the bathing time (within 60 s) in the antisolvent of tBuOH:EA also does not affect the device performance significantly (Fig. 3c). The wide processing window of tBuOH:EA is also confirmed by uniform morphology of perovskite layer fabricated upon various operation conditions (Supplementary Fig. 9).

PSCs fabricated using tBuOH:EA as an antisolvent displayed high PCE under optimized condition (Amount of DMSO = 1.5 eq. to FAPbI_3_, bathing time = 10 s, spin-coating duration at 5000 rpm = 10 s). The champion PCE of rigid, planar PSCs made on indium tin oxide (ITO) substrate and using SnO_2_ as an electron-transporting layer (ETL) is 22.2% in reverse scan, and 21.95% on average (Fig. 3d and Table 1). Mesoporous-structured PSCs based on a blocking- and mesoporous TiO_2_ as ETL with fluorine-doped tin oxide (FTO) substrate were also fabricated (Fig. 3d). The mesoporous device using FAPbI_3_ from bathing in tBuOH:EA showed a PCE of 23.5% in reverse scan, and 22.9% on average. Dripping of tBuOH:EA was also effective as bathing, as proven by the similar PCE (Supplementary Fig. 10). Consequently, the use of tBuOH:EA as antisolvent during the bathing is expected to guarantee a high PCE of PSCs with wide processing window for the R2R process.

**Table 1:** Summarized photovoltaic parameters of the devices fabricated in this study.

	Samples	*V*_oc_ [V]^a^	*J*_sc_ [mA/cm^2^]^a^	Fill factor [%]^a^	Efficiency [%]^a^
Rigid device made by using antisolvent bathing^b^	FTO/bl-TiO_2_/mp-TiO_2_/perovskite/ Spiro-OMeTAD/Au	1.17 (1.13)	24.8 (24.7)	81.0 (79.8)	23.5 (22.3)
	ITO/SnO_2_/perovskite/ Spiro-OMeTAD/Au	1.13 (1.14)	23.4 (23.4)	83.7 (81.3)	22.2 (21.7)
Table-top gravure-printed flexible devices^c^	Gravure-printed SnO_2_ & perovskite/ Spin-coated Spiro-	1.14 (1.10)	22.1 (22.1)	75.5 (73.3)	19.1 (17.8)
	Gravure-printed SnO_2_ & perovskite/ Shear-coated Spiro-	1.06 (−)	22.5 (−)	74.3 (−)	17.8 (−)
	Gravure-printed SnO_2_ & perovskite/ Gravure-printed Spiro-	1.02 (−)	22.5 (−)	73.5 (−)	17.1 (−)
R2R gravure-printed flexible devices^d^	R2R Gravure-printed SnO_2_ & perovskite/ Spin-coated Spiro-	1.02 (−)	22.0 (−)	74.4 (−)	16.7 (−)
	R2R Gravure-printed SnO_2_ & perovskite/ Table-top Shear-coated Spiro-	1.01 (−)	21.9 (−)	73.6 (−)	16.5 (−)
	R2R Gravure-printed SnO_2_ & perovskite/ Table-top Gravure-printed Spiro-	0.99 (0.94)	21.9 (21.8)	72.1 (71.2)	15.6 (14.6)
	R2R Gravure-printed SnO_2_ & perovskite/ Table-top Gravure-printed P3HT	1.01 (0.99)	20.9 (20.7)	66.3 (67.5)	14.0 (13.8)
	R2R Gravure-printed SnO_2_ & perovskite/ R2R Gravure-printed P3HT	0.96 (0.97)	20.9 (20.8)	68.1 (65.9)	13.8 (13.3)

^a^Champion values obtained from a reverse scan. The values in the parenthesis are obtained from a forward scan.

^b^Fig. 3d.

^c^Fig. 4d, e.

^d^Fig. 6d and Supplementary Fig. 20.

### Table-top gravure-printing of flexible PSCs at lab-scale

Various scalable processes have been applied to replace spin-coating. The coating methods including bar, slot-die, or blade coating have been employed to deposit perovskite layers^11,12,16,22,23,37–40^. These coating methods have an advantage of high-throughput deposition of desired layer, but methods require an additional patterning process after deposition. On the other hand, printing methods allows spontaneous formation of layers with desired shape and size with high resolution^41,42^. PSCs deposited by inkjet printing have recently shown a PCE of above 20%, but the deposition speed of inkjet printing is relatively low^41^. Gravure printing is a printing method that can combine the advantages of both methods, leading to spontaneous, high-throughput deposition of desired layer with arbitrary shape and size^15,18,19^. For the R2R process, which requires both high-throughput deposition and high resolution, gravure printing is a suitable process to use.

**Fig. 4:**
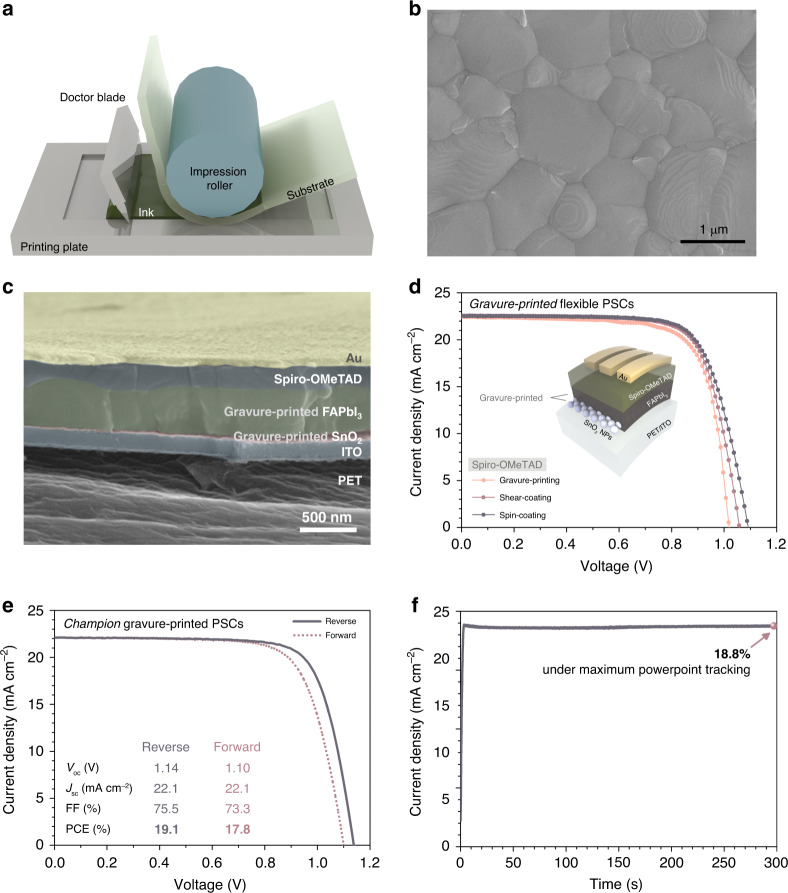
Table-top gravure-printing of multi-cation flexible PSCs. **a** Schematic illustration of table-top gravure printing. **b** SEM image of perovskite film fabricated by table-top gravure printing and subsequent bathing in tBuOH:EA. **c** Cross-sectional SEM image of flexible PSCs made by table-top gravure printing. Each layer was colored differently to distinguish it from the others. FAPbI_3_ indicates (FAPbI_3_)_0.95_(MAPbBr_3_)_0.05._ (inset) Diagram illustrating the structure of device used in this study. **d** Representative J-V curves of gravure-printed PSCs with Spiro-OMeTAD. Spiro-OMeTAD was deposited by spin-coating (indigo), shear-coating (purple) or gravure-printing (salmon). **e** A J-V curve of champion device fabricated by gravure-printing. **f** Efficiency of the champion devices measured by MPPT technique.

Table-top gravure printing of FAPbI_3_-based PSCs was conducted as depicted in Fig. 4a, prior to R2R fabrication at pilot-scale. A printing plate is located at the bottom, with a designed pattern having a specific engraving density to control transfer volume of ink. Printing ink is placed on the printing plate and scribed by a doctor blade to be fit in the engraved pattern. The ink is transferred to the substrate by pressure exerted with a roller. SnO_2_ nanoparticles (NPs) were uniformly gravure-printed on ITO/PET substrates by reducing the surface tension of the ink^15^ (Supplementary Fig. 11). (FAPbI_3_)_0.95_(MAPbBr_3_)_0.05_ films were uniformly formed on top of the SnO_2_ layer in large-area by gravure printing and subsequent bathing in tBuOH:EA (Supplementary Fig. 12). The perovskite film is formed with large, densely packed grains, and a highly crystalline nature without PbI_2_, as confirmed by the SEM image and XRD spectrum (Fig. 4b and Supplementary Fig. 13).

Spiro-OMeTAD as an HTL was deposited on the top of the perovskite/SnO_2_, all layers of which are formed by gravure printing. The overall device structure is PET/ITO/SnO_2_ NPs/(FAPbI_3_)_0.95_(MAPbBr_3_)_0.05_/Spiro-OMeTAD/Au (Fig. 4c). The thicknesses of the layers were 25, 450, 210, and 70 nm for SnO_2_, (FAPbI_3_)_0.95_(MAPbBr_3_)_0.05_, Spiro-OMeTAD, and Au, respectively. Although we previously demonstrated successful gravure printing of Spiro-OMeTAD on MAPbI_3_, gravure-printed Spiro-OMeTAD on top of FA-based perovskite was not as effective as printing on MAPbI_3_ because of the rough surface of the FA-based perovskite layer and the low viscosity of the Spiro-OMeTAD solution^15^ (Fig. 4d and Table 1). Instead, Spiro-OMeTAD was deposited by solution shear-coating, which is basically receding meniscus coating with a low-angle blade^22,38^ (Supplementary Fig. 14). The low viscosity and highly volatile characteristic of the Spiro-OMeTAD solution make it more suitable for solution shear-coating than for gravure-printing. PSCs fabricated by all scalable processes including gravure printing of SnO_2_/(FAPbI_3_)_0.95_(MAPbBr_3_)_0.05_ and shear-coating of Spiro-OMeTAD showed PCE of 17.8%.

The champion PCE of flexible PSCs based on gravure-printed SnO_2_/(FAPbI_3_)_0.95_(MAPbBr_3_)_0.05_ and spin-coated Spiro-OMeTAD is 19.1% in reverse scan and 18.45% on average, which is comparable to the PCE of the best flexible PSCs based on a spin-coating^43–45^ (Fig. 4e and Table 1). The PCE obtained from maximum power point tracking measurement (MPPT) is 18.8%, which is very close to the PCE obtained from the current density-voltage scan in the reverse direction. Short-circuit current density (*J*_sc_) was well-matched with the value obtained from external quantum efficiency (EQE) measurement (Supplementary Fig. 15). The average performance of gravure-printed PSCs is open-circuit voltage (*V*_oc_) of 1.10 V, *J*_sc_ of 22.1 mA cm^−2^, fill factor (FF) of 73.0%, and PCE of 17.8% (Averaged from 24 devices. Supplementary Figs. 16 and 17).

### Pilot-scale R2R gravure-printing of flexible PSCs

We attempt to extend our approach based on antisolvent-bathing process for gravure-printed flexible PSCs into R2R fabrication at pilot-scale, which still remains several issues to be delicately considered, such as the large-scale preparation of the perovskite ink, the feasible short processing time, the choice of the cost-effective HTL, etc.

**Fig. 5:**
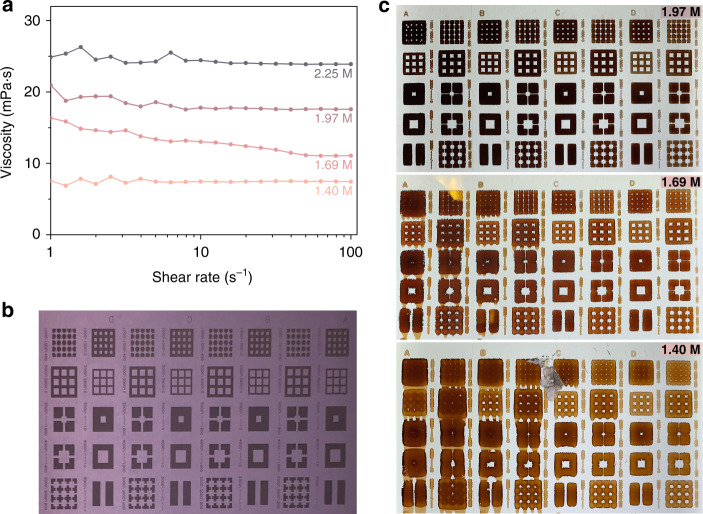
Modulation of perovskite inks and impact on the resolution of gravure-printed pattern. **a** Viscosity of perovskite precursor solutions having different concentrations. Images of (**b**) printing plate and (**c**) printed perovskite patterns on PET substrate with different concentrations.

For the perovskite ink, the viscosity of the precursor solution was optimized to deposit a proper thickness with high resolution in R2R printing and achieve a sophisticated pattern. To print layers with high resolution, the viscosity of the ink should be increased, or the transfer volume should be smaller to prevent ink from spreading too much. The former is our best choice with respect to cost-effectiveness and reliability. We prepared a highly concentrated solution of perovskite precursor and found that the viscosity of the solution dramatically could be increased from 7.4 to 23.9 mPa s at 100 s^−1^ (Fig. 5a). We investigated the viscosity effect of perovskite precursor solution on the resolution of gravure-printed film by demonstrating a sophisticated pattern of the perovskite film (Fig. 5b and c). Upon increasing the solution viscosity, the resolution of resulting patterns is improved. The edges of patterns look sharper and tailing is reduced. In consideration of pattern definition and thickness of the film, we determine that the optimum concentration of perovskite solution for R2R is 1.69 M.

**Fig. 6:**
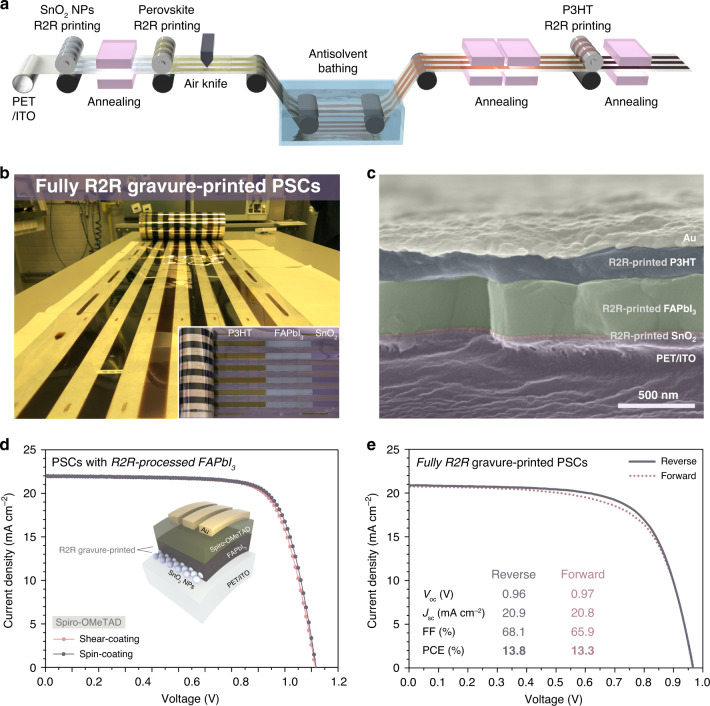
Fully R2R gravure printing of PSCs except electrodes. **a** Diagram showing R2R processing for the fabrication of flexible PSCs. **b** Photograph of fully R2R-processed PSCs. (inset) Image of a R2R-processed roll showing constituent layers of PSCs after removing each layer manually. **c** Cross-sectional SEM image of fully R2R printed PSCs. Each layer was given a different color. **d** J-V curves of champion device made from R2R-processed SnO_2_/perovskite and Spiro-OMeTAD (shear-coating: purple, spin-coating: indigo). **e** The J-V curves of fully R2R gravure-printed PSCs. FAPbI_3_ denotes (FAPbI_3_)_0.95_(MAPbBr_3_)_0.05_.

R2R production of flexible PSCs at pilot-scale was successfully conducted via sequential R2R gravure printing of SnO_2_ NPs, perovskite, and P3HT as illustrated in Fig. 6a. In this demonstration of R2R, P3HT was selected as HTL because of the lower cost and better printability as previously shown in OPVs, even though the device using Spiro-OMeTAD exhibited higher efficiency^7,17,18^. SnO_2_ NPs were R2R gravure-printed followed by short-term annealing. Perovskite precursor solution was first R2R gravure-printed; then, the as-deposited film was exposed to air-blowing from an air knife to adjust the amount of excess processing solvent, and passed through the tBuOH:EA bath to convert phase to intermediate phase, and finally annealed in a hot oven to be crystallized. P3HT was also R2R gravure-printed, and thermally annealed. The whole procedure was recorded as Supplementary Movie 1 and Supplementary Fig. 18.

As a result, we successfully attained a 30-cm-wide, 100-m-long roll of fully R2R-processed PSCs except for the Au metal (Fig. 6b). Uniform, pin-hole free films of SnO_2_, perovskite, and P3HT layers were successfully deposited by R2R gravure printing (Supplementary Fig. 19). The roll was cut into sheets and Au electrode was formed by evaporation. The structure of the device is shown in Fig. 6c. The thicknesses of the layers are 40, 400, 180, and 100 nm for SnO_2_, (FAPbI_3_)_0.95_(MAPbBr_3_)_0.05_, P3HT, and Au, respectively.

The quality of R2R gravure-printed (FAPbI_3_)_0.95_(MAPbBr_3_)_0.05_ was verified by the fabrication of flexible PSCs based on R2R gravure-printed SnO_2_/(FAPbI_3_)_0.95_(MAPbBr_3_)_0.05_ and Spiro-OMeTAD HTL (Fig. 6d and Table 1). Spiro-OMeTAD was either spin-coated or solution shear-coated, the same as the table-top printed perovskite. PSCs composed of R2R printed SnO_2_/(FAPbI_3_)_0.95_(MAPbBr_3_)_0.05_ and spin-coated Spiro-OMeTAD result in a high PCE of 16.7%, which is the best efficiency among PSCs from R2R-processed perovskite^13,14,46,47^. Solution shear-coating of Spiro-OMeTAD was also applied to the R2R-processed perovskite, resulting in a device with 16.5% of PCE, which value is almost the same to that from spin-coated HTL. We also attempted to apply gravure printing to deposit Spiro-OMeTAD and P3HT as a HTM on the top of R2R gravure-printed SnO_2_/(FAPbI_3_)_0.95_(MAPbBr_3_)_0.05,_ which resulted in slightly lower performance for Spiro-OMeTAD than other processing methods (Supplementary Fig. 20 and Table 1). Fully R2R gravure-printed PSCs showed PCE of 13.8% from scan in reverse direction, and 13.55% on average when P3HT was used as HTL. This is the highest PCE ever reported for fully R2R-processed PSCs^11,16^ (Fig. 6e and Table 1). Note that the P3HT-based devices fabricated by table-top or R2R gravure printing showed almost identical efficiencies, revealing the effectiveness of R2R gravure-printing at a pilot scale (Supplementary Fig. 20 and Table 1). Average PCE from fully R2R-processed devices was 12.4%, with relatively narrow distribution, which illustrates the reliable production of PSCs by sequential R2R gravure printing (Supplementary Fig. 21). The stability of the fully R2R-processsed PSCs was investigated by fabrication of the PSCs from the different part of the as-prepared roll (PET/ITO/SnO_2_/(FAPbI_3_)_0.95_(MAPbBr_3_)_0.05_/P3HT) for every few days, by randomly cutting into sheets and evaporating Au electrodes (Supplementary Fig. 22). The PSCs made after almost 40 days from the production of the roll showed similar levels of PCE, indicating the high storage stability and reliability of the PSCs made by R2R gravure printing and the process itself. The large-area applicability and reliability of the R2R gravure printing was confirmed by a slight decrease in PCE of large-area (1 cm^2^) devices compared to that of the smaller devices (Supplementary Fig. 23). We believe that the device performance for fully R2R-processed PSCs would be obviously enhanced by incorporation of new HTL suitable for R2R process. Our approach using eco-friendly antisolvent bathing with a wide processing window can be universally applied into gravure-printed flexible PSCs at lab & pilot-scale, thus offering an effective way toward R2R fabrication of flexible PSCs with high efficiency.

## Discussion

In conclusion, we demonstrated R2R-processed flexible PSCs at pilot scale through gravure-printing, antisolvent bathing, and subsequent annealing process. As an eco-friendly anti-solvent, we choose tert-butanol (tBuOH), which was used for antisolvent-bathing. The mixed co-solvent of tBuOH:EA offers a formation of a highly crystalline and uniform perovskite film with wide processing window. As a result of antisolvent-bathing, we achieved a champion PCE of 23.5% (in reverse scan) from the glass-used spin-coated device. The gravure printing of FAPbI_3_-based perovskite layers was conducted and the best PCE of 19.1% was achieved in gravure-printed flexible PSCs, which is one of the highest PCE of flexible PSCs to the best of our knowledge. As an extended work, we attempted to fabricate full R2R gravure printing of flexible PSCs except for top electrodes at a pilot-scale via process optimization to successfully demonstrate a manufacturing of 100-meter-long roll and a high PCE exceeding 16% and 13%, when Spiro-OMeTAD and P3HT was used as HTL, respectively. We believe that this work will pave a new way for high throughput, low-cost production of flexible PSCs at larger scale via R2R processes in the near future.

## Methods

### Chemicals

For perovskite layer, lead (II) iodide (99.999%, ultra dry) from Alfa Aesar, lead (II) bromide (perovskite precursor grade) from TCI, formamidinium iodide (FAI, Greatcell Solar), methylammonium iodide (MAI, Greatcell Solar), methylammonium bromide (MABr, Greatcell Solar) and methylamine hydrochloride (≥98%, MACl, Sigma-Aldrich)) were used. Tin (IV) oxide solution (15% in H_2_O) was purchased from Alfa Aesar. Titanium diisopropoxide bis(acetylacetonate) (75 wt% in isopropanol), Lithium bis(trifluoromethanesulfonyl)imide (≥99%, Li-TFSI) and 4-tert-butyl pyridine (98%, TBP) were purchased from Sigma-Aldrich. N2,N2,N2′,N2′,N7,N7,N7′,N7′-octakis(4-methoxyphenyl)-9,9′-spirobi[9H-fluorene]-2,2′,7,7′-tetramine (99%, Spiro-OMeTAD), and Tris(2-(1H -pyrazol-1-yl)-4-tert-butylpyridine) - cobalt(III) Tris(bis(trifluoromethylsulfonyl)imide)) (>99%, FK209 Co(III) TFSI) were purchased from LumTec. Mesoporous TiO_2_ paste was purchased from ShareChem. Poly(3-hexylthiophene) (#4002-E or #4002-EE) was purchased from Rieke Metals. All the other chemicals including organic solvents were purchased from Sigma-Aldrich if they are not noted.

### Rigid device fabrication

For the TiO_2_-based devices, a dense TiO_2_ layer was deposited on a fluorine-doped SnO_2_ subtrate (FTO, Pilkington, TEC8) by spray pyrolysis of titanium diisopropoxide bis(acetylacetonate) solution in ethanol at 450 °C. Mesoporous TiO_2_ layer was then deposited on the top of the blocking TiO_2_ layer by spin-coating of diluted solution (2-methoxyethanol:terpineol = 3.5:1 by v/v) of TiO_2_ paste followed by thermal annealing at 500 °C for 1 h. Li-TFSI solution in acetonitrile was spin-coated on the TiO_2_ layer, then annealed at 500 °C. For SnO_2_ layer, diluted SnO_2_ dispersion (1:5 in water by v/v) was spin-coated on the ITO substrate and annealed at 100 °C. A perovskite solution was prepared by 1.26 mmol of FAI and PbI_2_, and 0.0632 mmol of MABr, PbBr_2_, and MACl into 0.8 mL of DMF and 0.15 mL of DMSO. The perovskite layer was formed by a multi-step spin-coating of the precursor solution at 500, 1000, and 5000 rpm. After spin-coating, the substrate was dipped immediately in the bath filled with antisolvent. The resulting substrate was blown by compressed air to remove remnant antisolvent on the substrate, then placed on the hot plate at 100 °C. For the champion TiO_2_-based device, butylammonium iodide dissolved in IPA was spin-coated on the top of perovskite layer at 4000 rpm for 30 s and annealed at 100 °C for 5 min. A Spiro-OMeTAD was dissolved in chlorobenzene (0.0909 g mL^−1^) with 23 μL of Li-TFSI/Acetonitrile (540 mg ml^−1^), 39 μL of TBP, and 10 μL of FK209/acetonitrile (0.376 g ml^−1^). The Spiro-OMeTAD solution was spin-casted at 2000 rpm for 30 s. The devices were completed by thermal evaporation of Au with shadow mask to define cell area.

### Fabrication of gravure printed, flexible PSCs at a lab-scale

A ITO-coated PET roll (Eastman Flexvue OC50) was used as a substrate. The ITO was selectively patterned by rotary screen printing of an etching paste. (HiEP-300, P & P Solution Co., Ltd) The roll was used without further purification. For the laboratory-scale printing tests, ITO/PET roll was cut into individual sheets. The gravure printing at a laboratory-scale was conducted by using a table-top gravure-printing machine (Labratester, Norbert Schläfli Maschinen). SnO_2_ nanoparticles were diluted by mixture of water and IPA (4:1 by v/v) to reduce surface tension, then gravure printed by a printing plate with an engraved pattern of 120 lines cm^−1^, at a speed of 18 m min^−1^. A resulting film was annealed in a hot oven at 120 °C for 5 min. For a perovskite layer, 0.5 mL of the precursor solution was dispensed on the printing plate with an engraved pattern having a line density of 120 lines cm^−1^, then printed at a speed of 18 m min^−1^. The freshly printed film was dried for 1 min, then immersed in a bath filled with an antisolvent mixture of tert-butyl alcohol and EA (7:3 by v/v). After 30 s of bathing, the film was blown by compressed air, then annealed in the oven at 120 °C for 30 min. A Spiro-OMeTAD solution with additives was formed by either spin-coating, gravure-printing, or shear-coating. For the spin-coating, the Spiro-OMeTAD solution was spin-coated at 2000 rpm for 30 s. For the gravure-printing, the Spiro-OMeTAD solution was dispensed at the printing plate having a line density of 100 lines cm^−1^, then printed at a speed of 18 m min^−1^. Shear-coating of Spiro-OMeTAD was conducted by using a table-top multicoater (PMC-200, PEMS, Republic of Korea) equipped with glass blade. A solution was placed on the top of the substrate having a gap with a blade of 100 μm, then coated by a rate of 10 mm s^−1^. Au electrodes were evaporated by thermal evaporation with a shadow mask to define the cell area.

### R2R printing of PSCs

R2R gravure printing was conducted by using a custom-built pilot-scale R2R printing machine^15^. A 100 m-long PET roll with patterned ITO electrode was used as a substrate. The roll was Ar/N_2_ plasma-treated, then SnO_2_ nanoparticles were roll-to-roll gravure printed at a speed of 8 m min^−1^ followed by annealing at 120 °C for 30 s by hot air oven. The perovskite precursor solution was made to have a concentration of 1.69 M, and R2R gravure printed after plasma treatment at a speed of 3 m min^−1^. After printing of wet-film, the roll was blown by air knife to remove extra solvent from wet-film, then passed through a bath filled with the antisolvent mixture of tBuOH and EA for 10 s. Note that it is not necessary to substitute the antisolvent during R2R printing of a 100 meters-long roll. The extra amount of antisolvent was blown by second air knife equipped after the bath. The roll was annealed by passing through the hot air oven at 140 °C. A P3HT was dissolved in chlorobenzene/chloroform (8:2 by v/v) at a concentration of 55 mg ml^−1^, with Li-TFSI solution (540 mg ml^−1^ in acetonitrile) and TBP as dopants. P3HT was R2R gravure printed at a speed of 8 m min^−1^ and annealed at 80 °C for 30 s. The roll was cut into individual sheets and Au electrode was evaporated to complete the device structure.

### Characterization

The top-view and cross-sectional image of perovskite films and solar cells were obtained by using field emission scanning electron microscope (SEM, Mira 3 LMU, Tescan) operated at 20 kV. Physical dimensions from the images were obtained by using ImageJ software (U.S. National Institutes of Health, https://imagej.nih.gov/ij/). X-ray diffraction spectra were obtained by using Rigaku smartlab X-ray diffractometer. The J-V curves and MPPT tracking curve were obtained by using a solar simulator (Newport, Oriel Class A, 91195A) with voltage sourcemeter (Keithley 2400) under 100 mW cm^−2^ illumination with standard AM1.5G condition. Light intensity was calibrated Si-reference cell certified by the NREL, USA. The measurement was performed under ambient atmosphere, scanned in range from −0.2 to 1.2 V, with 10 ms of delay time. The samples were all masked with metal-mask having an active area of 0.096 cm^2^. The active area defined by metal mask was measured by using optical microscope. EQE was measured by using QUANTX-300 QE measurement system (Oriel Instruments). The Viscosity of solutions was measured by ARES-G2 Rheometer using 40 mm parallel plate geometry while retaining the temperature of solution at 25 °C. Fourier-transform Infrared (FT-IR) spectra was measured in ATR mode, by using compact FT-IR spectrometer ALPHA II (Bruker corporation). Time-resolved PL (TRPL) study was performed using an inverted-type scanning confocal microscope (MicroTime-200, Picoquant, Germany) with a 40× (air) objective. The lifetime measurements were performed at the Korea Basic Science Institute (KBSI), Daegu Center, Korea. A single-mode pulsed diode laser (470 nm with a pulse width of ~30 ps) was used as an excitation source. A dichroic mirror (490 DCXR, AHF), a 75 μm pinhole, and a single-photon avalanche diode (PDM series, MPD) were used to collect emission from the samples. Time-correlated single-photon counting technique was used to count emission photons. TRPL images consisted of 200 × 200 pixels were recorded using the time-tagged time-resolved data acquisition method.

### Reporting summary

Further information on research design is available in the Nature Research Reporting Summary linked to this article.

## Supplementary information

Supplementary Information

Description of Additional Supplementary Files

Supplementary Movie 1

Reporting Summary

## Data Availability

The data that support the findings of this study are available from the corresponding author upon reasonable request.
