# Supplementary material for: Roll-to-roll gravure-printed flexible perovskite solar cells using eco-friendly antisolvent bathing with wide processing window
Source: Nat Commun. 2020 Oct 13;11:5146. doi: 10.1038/s41467-020-18940-5 (PMC7555830; doi:10.1038/s41467-020-18940-5)
Supplement: Supplementary file 1 — Supplementary Information [file 41467_2020_18940_MOESM1_ESM.pdf]

Supplementary Information

Roll-to-roll gravure-printed flexible perovskite solar cells using eco-friendly antisolvent bathing with wide processing window

Young Yun Kim[1], *Tae-Youl Yang*[1,2], *Riikka Suhonen*[3], *Antti Kemppainen*[3], *Kyeongil Hwang*[1], *Nam Joong Jeon*[1], *Jangwon Seo*[1,*]

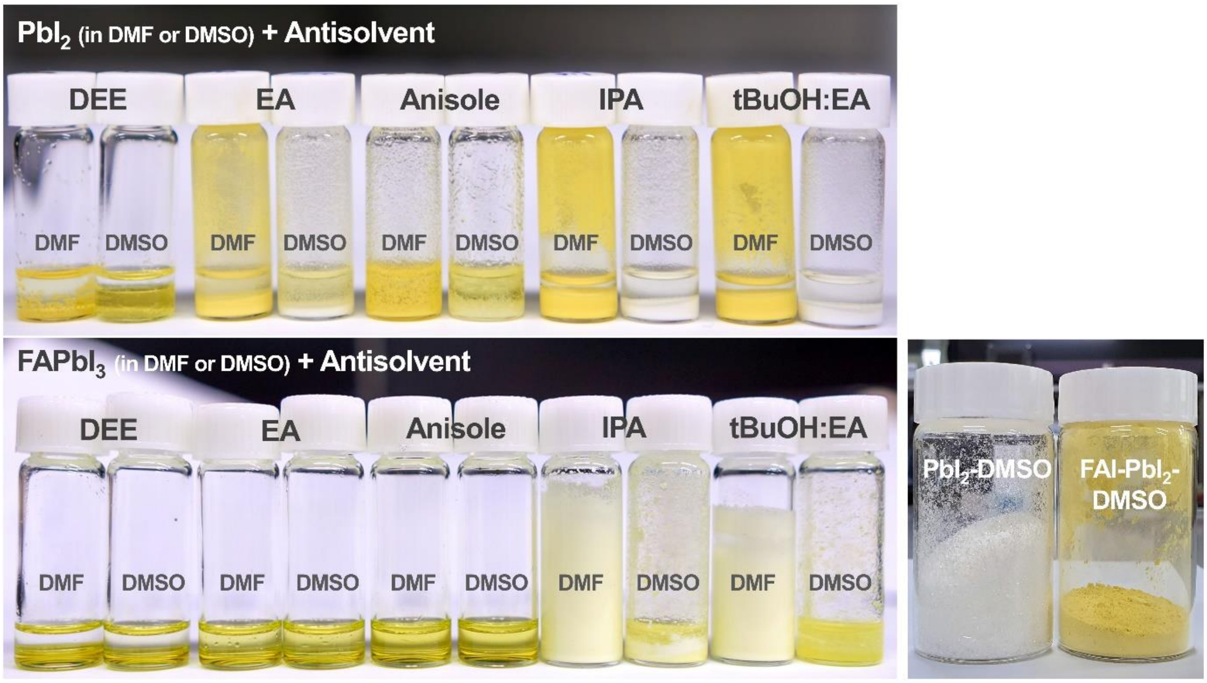

Supplementary Figure 1. Photographs of mixed dispersion of excess antisolvent and (top) PbI_2 or (down) $FAPbI_3$. (left) A photograph of PbI_2-DMSO and FAI-PbI_2-DMSO powders.

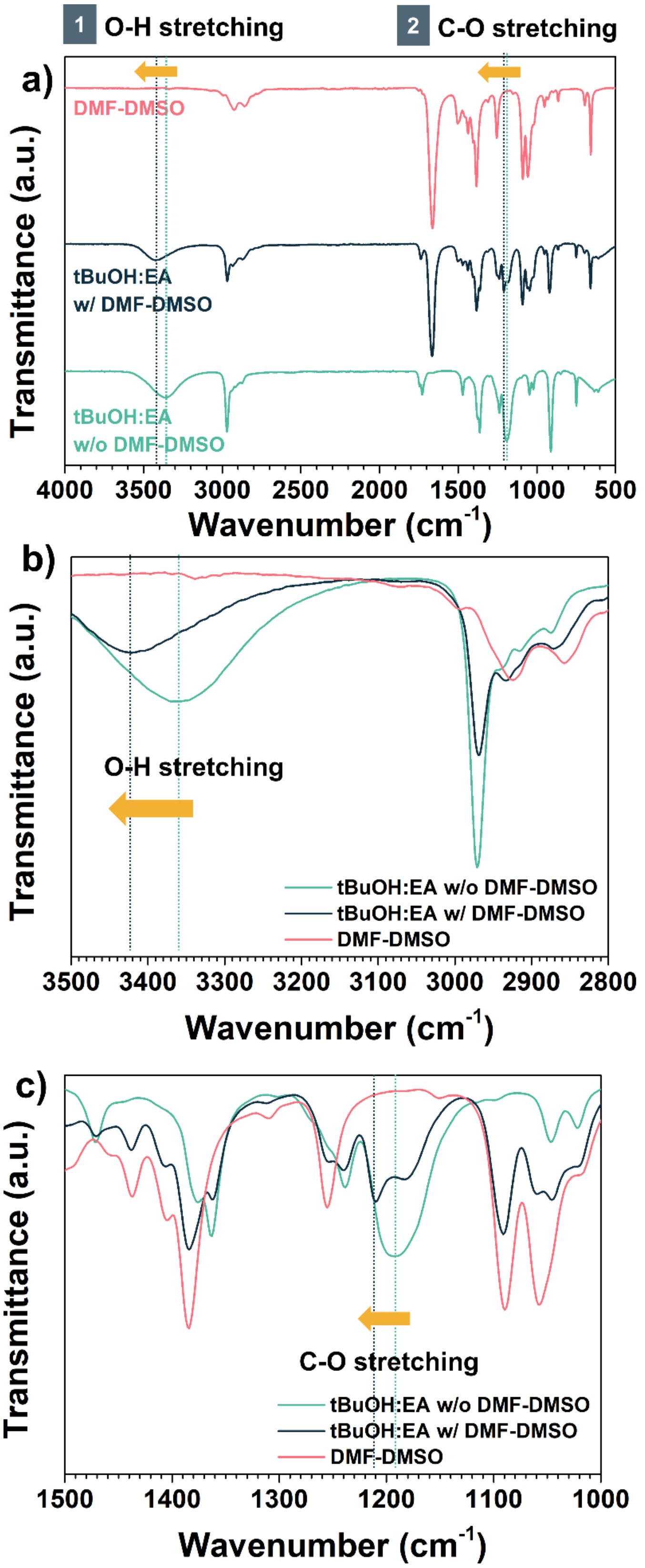

Supplementary Figure 2. a) IR spectra of DMF-DMSO, tBuOH:EA, and their mixture. b), c) Enlarged IR spectra for specific wavenumber.

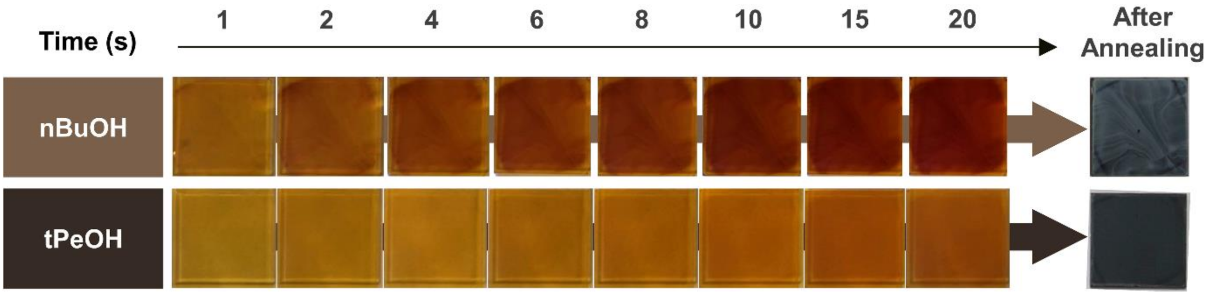

Supplementary Figure 3. Pictures representing time evolution of perovskite intermediate film during bathing in nBuOH or tPeOH.

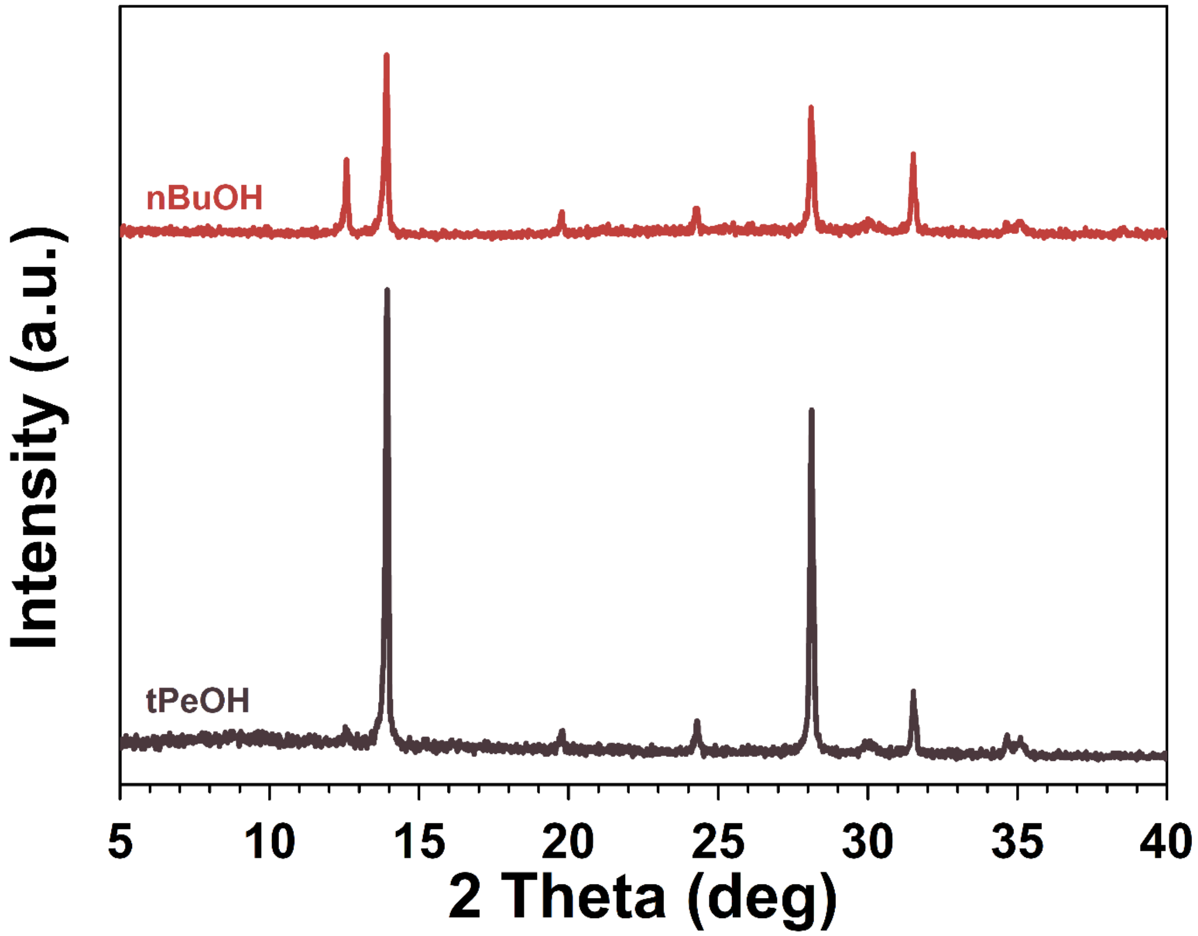

Supplementary Figure 4. XRD spectra of perovskite film made by bathing in nBuOH or tPeOH.

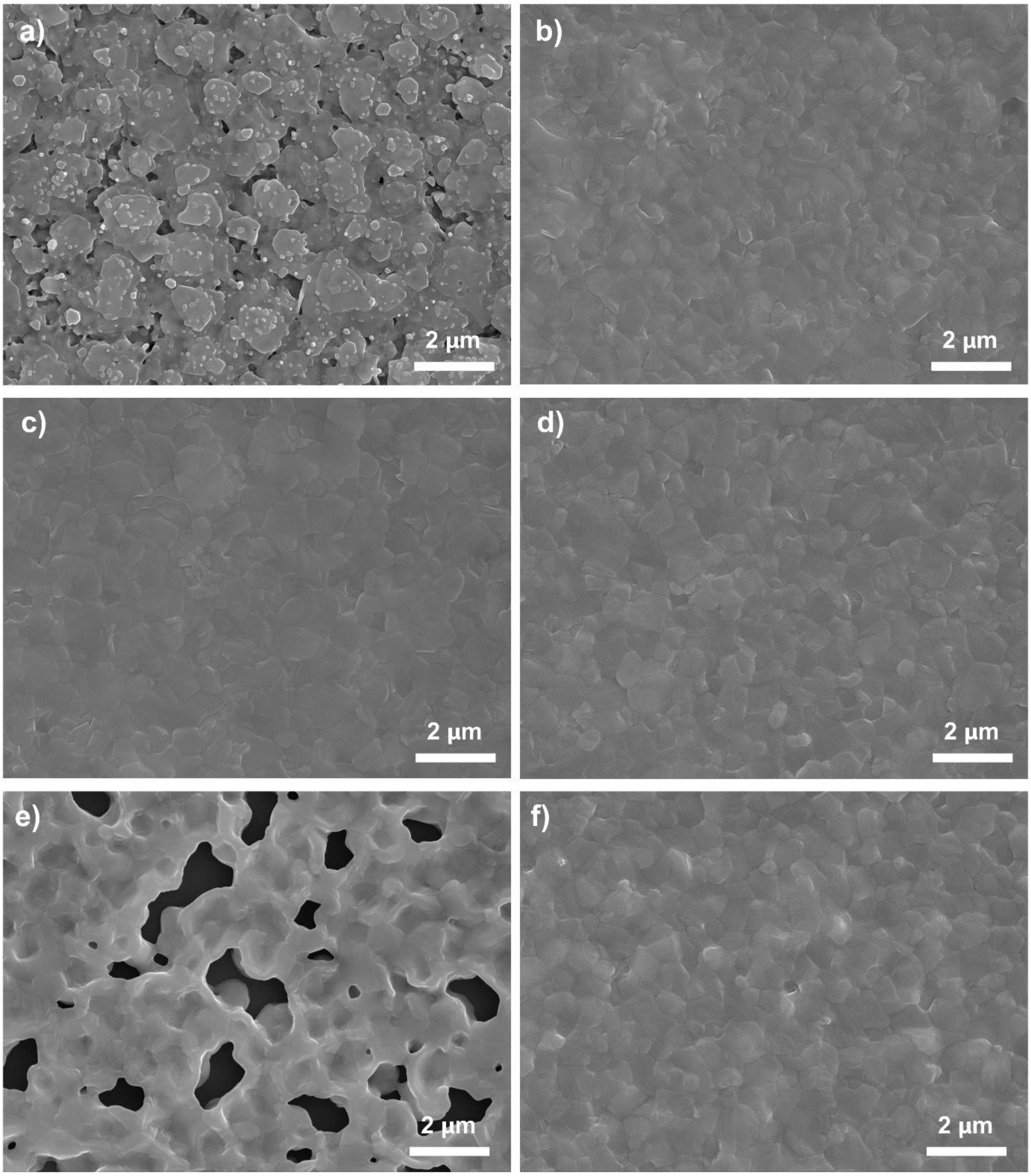

Supplementary Figure 5. SEM images of perovskite films fabricated through bathing in various antisolvents. a) EtOH, b) IPA, c) nBuOH, d) tPeOH, e) Anisole, f) EA.

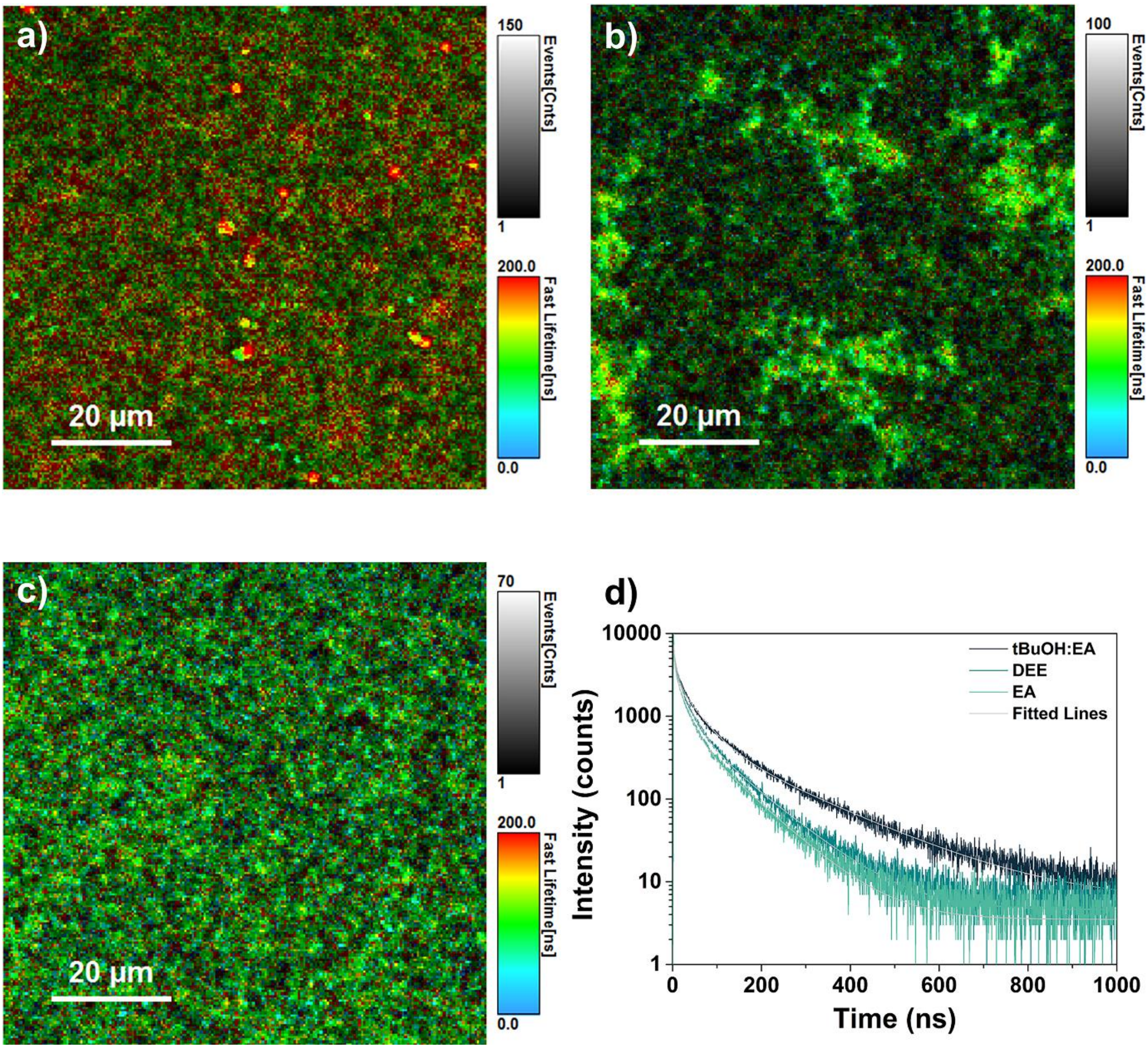

Supplementary Figure 6. FLIM images of the perovskite film made by using a) tBuOH:EA, b) DEE, and c) EA as an antisolvent. d) TRPL spectra of corresponding perovskite films obtained from FLIM images.

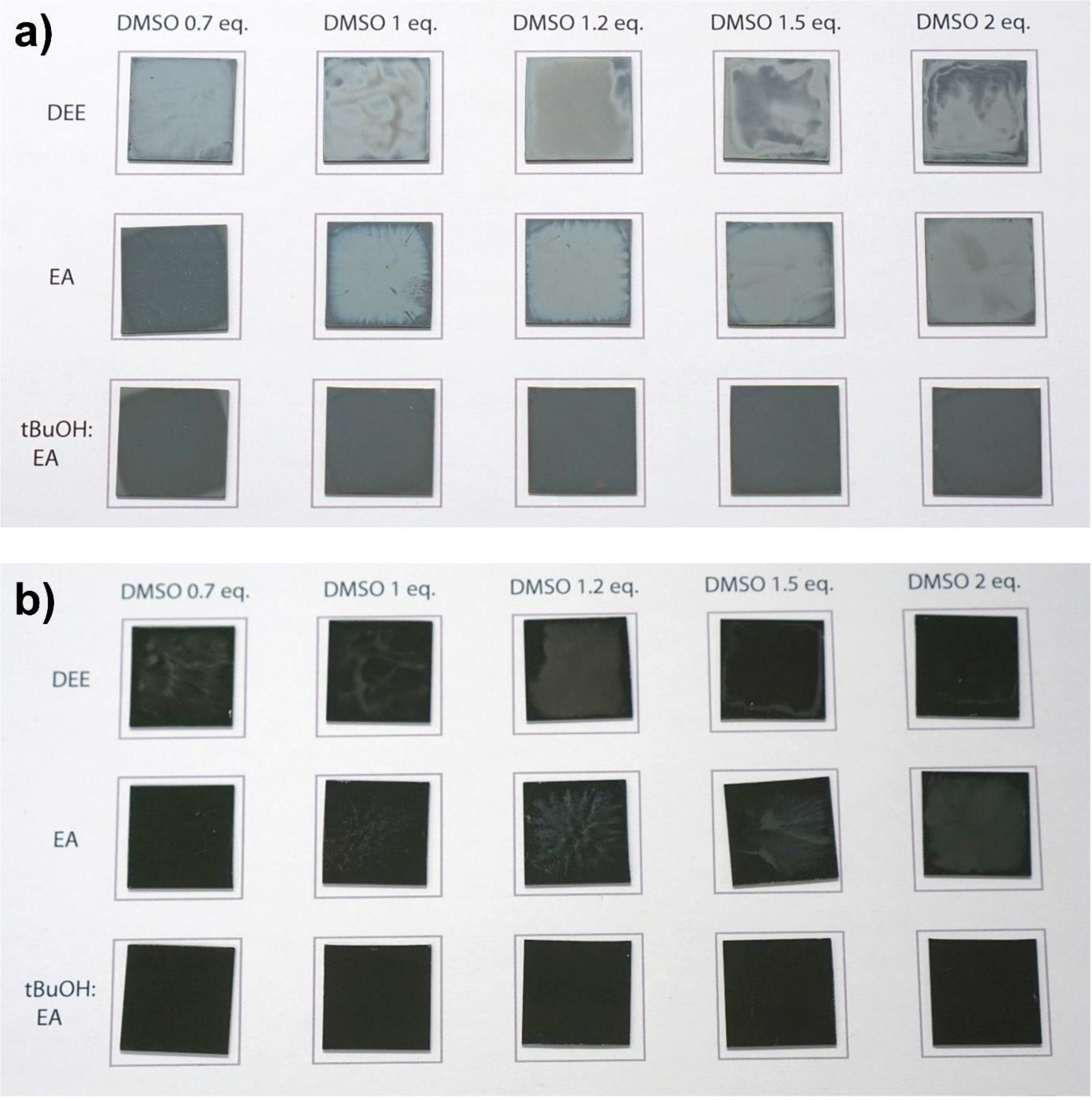

Supplementary Figure 7. a) Front-side, and b) back-side photographs of perovskite films made by using different antisolvents upon the different amount of DMSO in the precursor solution.

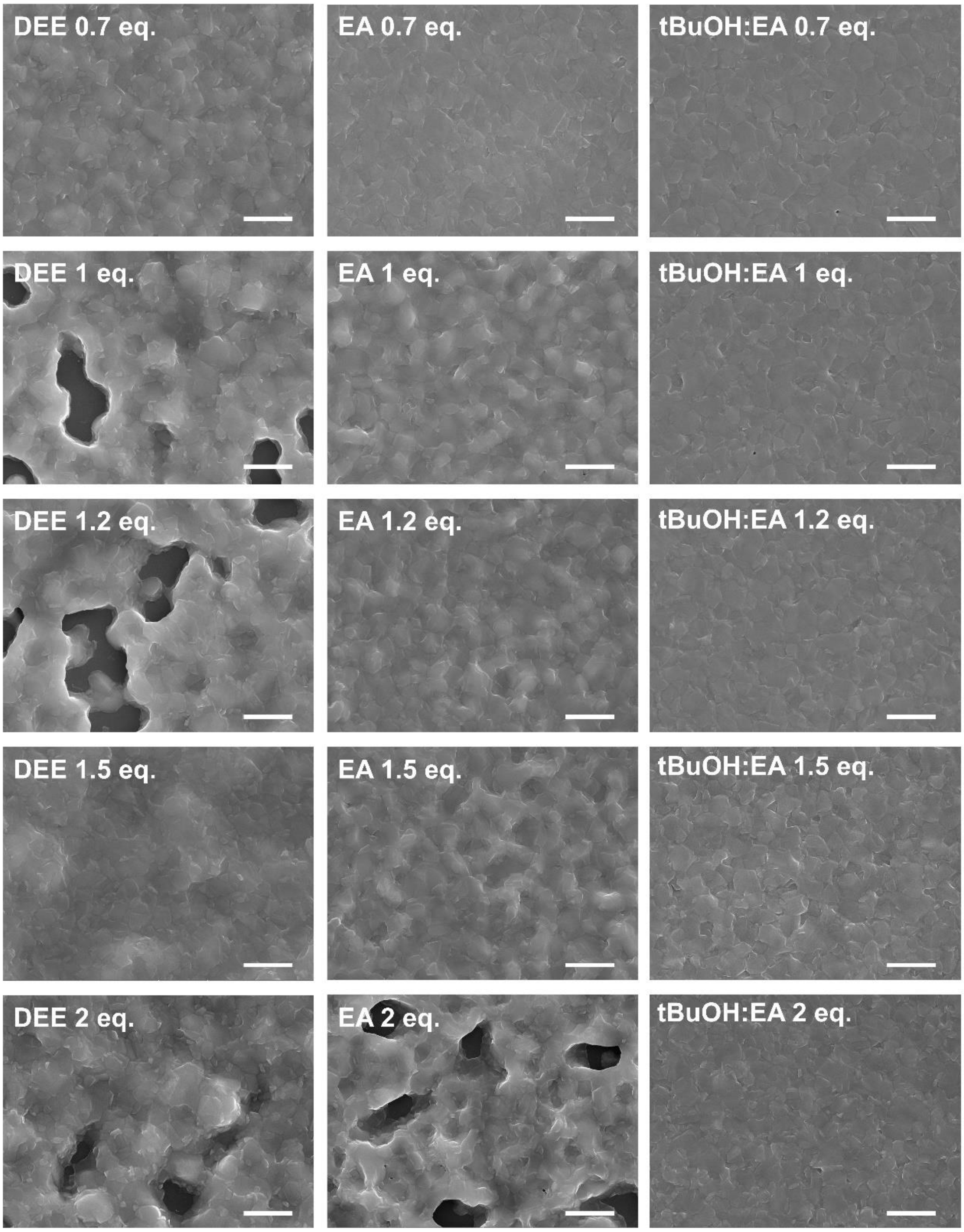

Supplementary Figure 8. SEM images of perovskite films made by using different antisolvents while varying the amount of DMSO in the precursor solution. The scale bar is 2 μm.

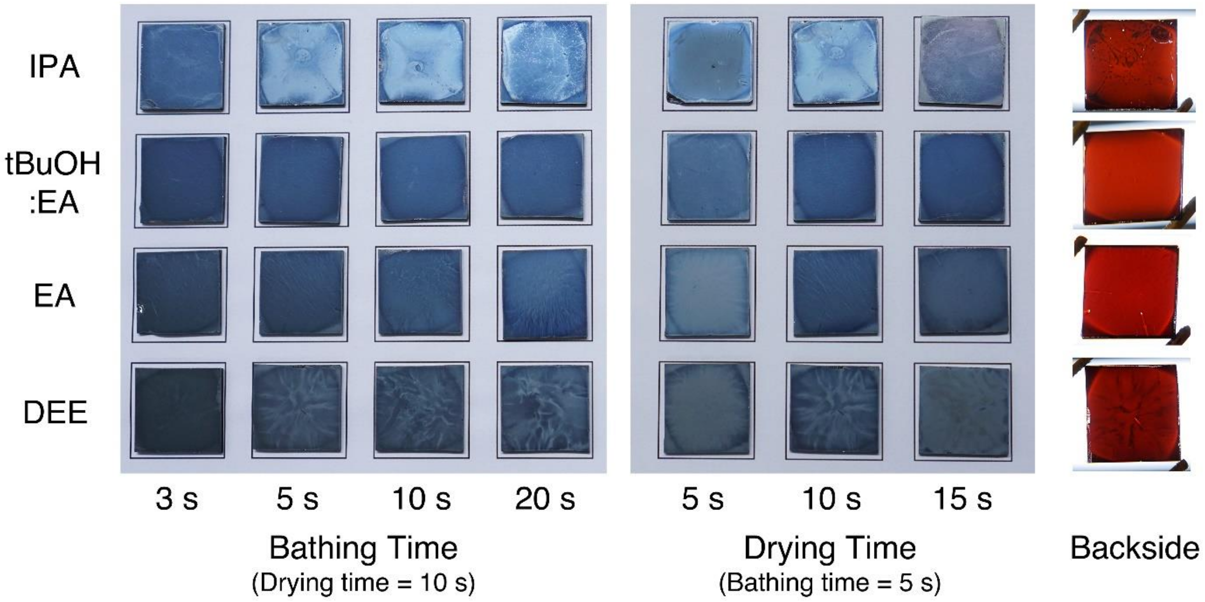

Supplementary Figure 9. Images of perovskite films obtained by antisolvent bathing under various processing conditions.

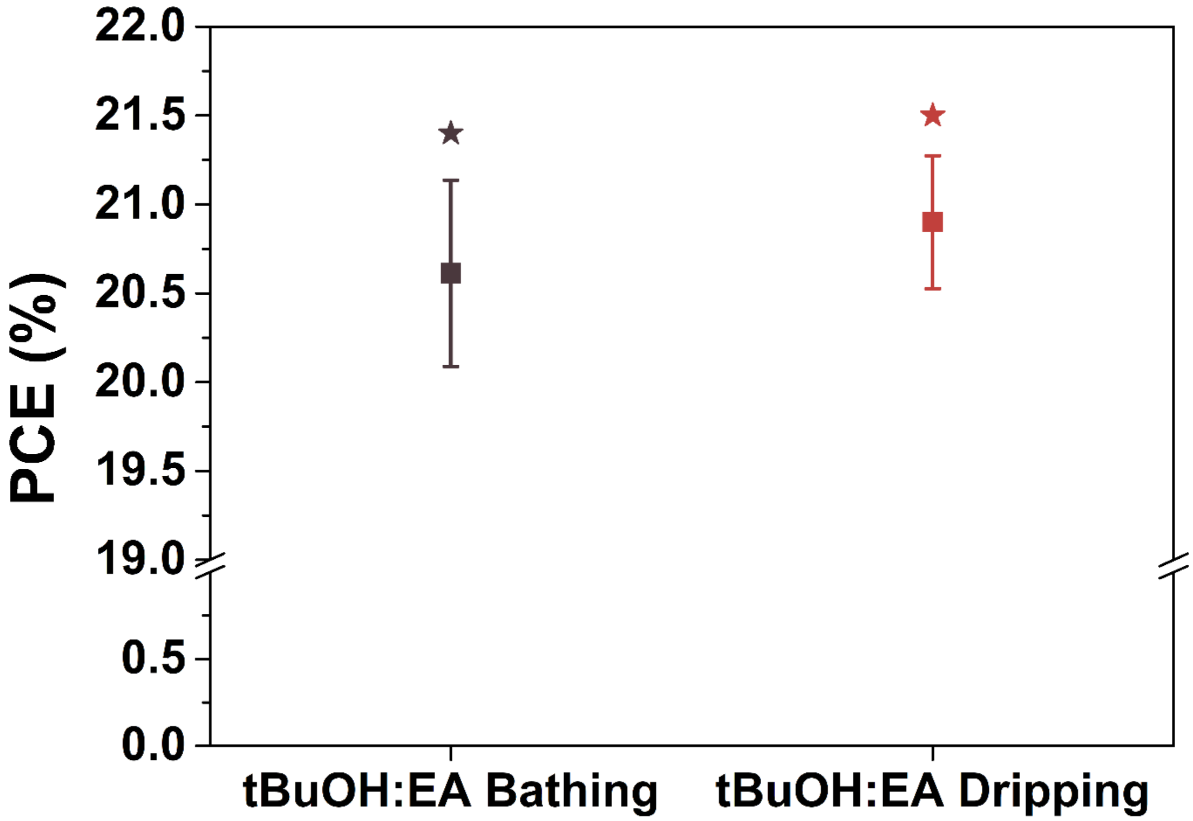

Supplementary Figure 10. The average (square symbol; bars indicate standard deviation) and champion (star symbol) PCE of PSCs based on a bathing or dripping of tBuOH:EA. The PCE was averaged from 12 devices.

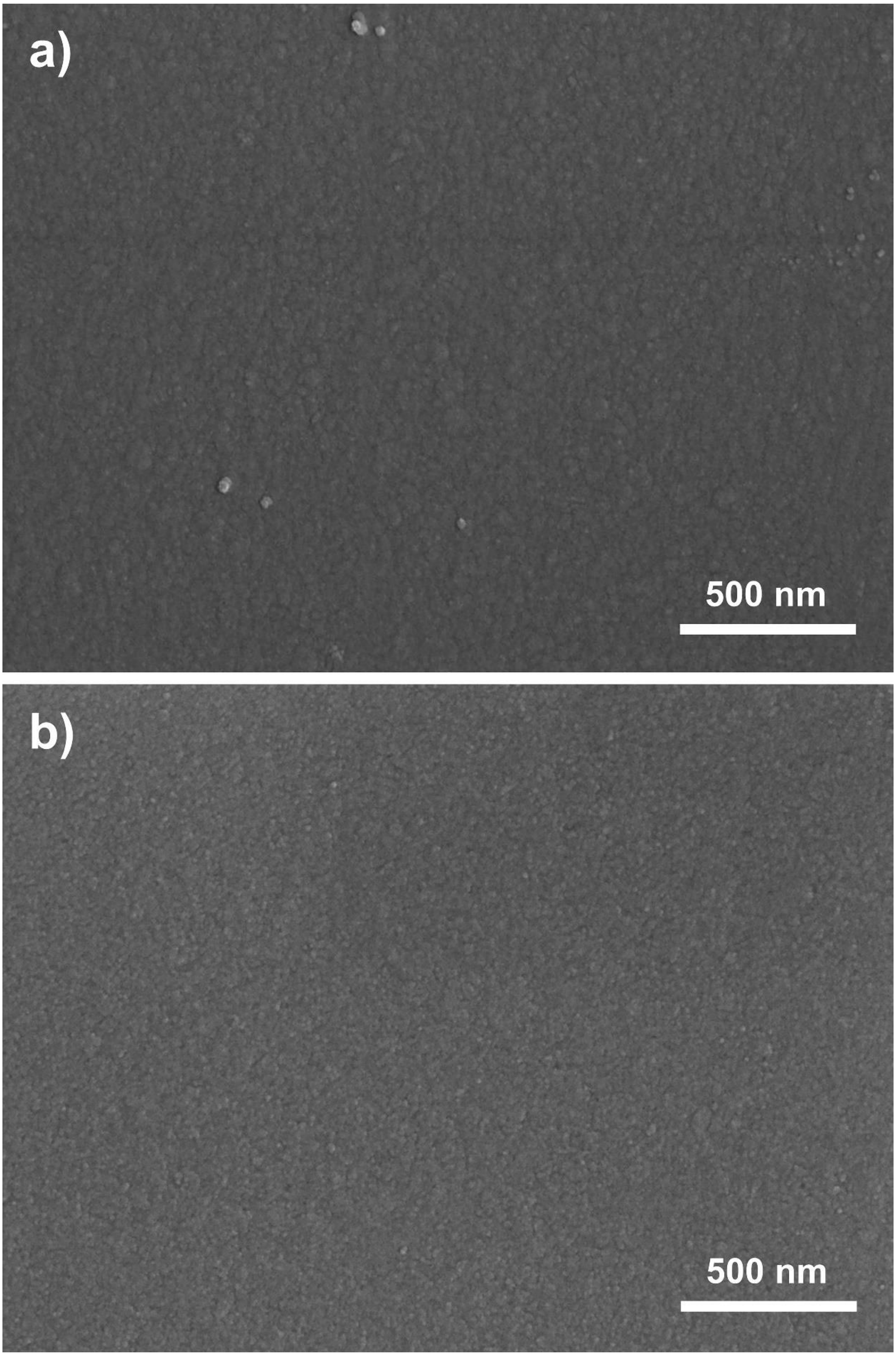

Supplementary Figure 11. Top-view SEM images of a) bare ITO and b) gravure-printed SnO_2 layer on the top of ITO.

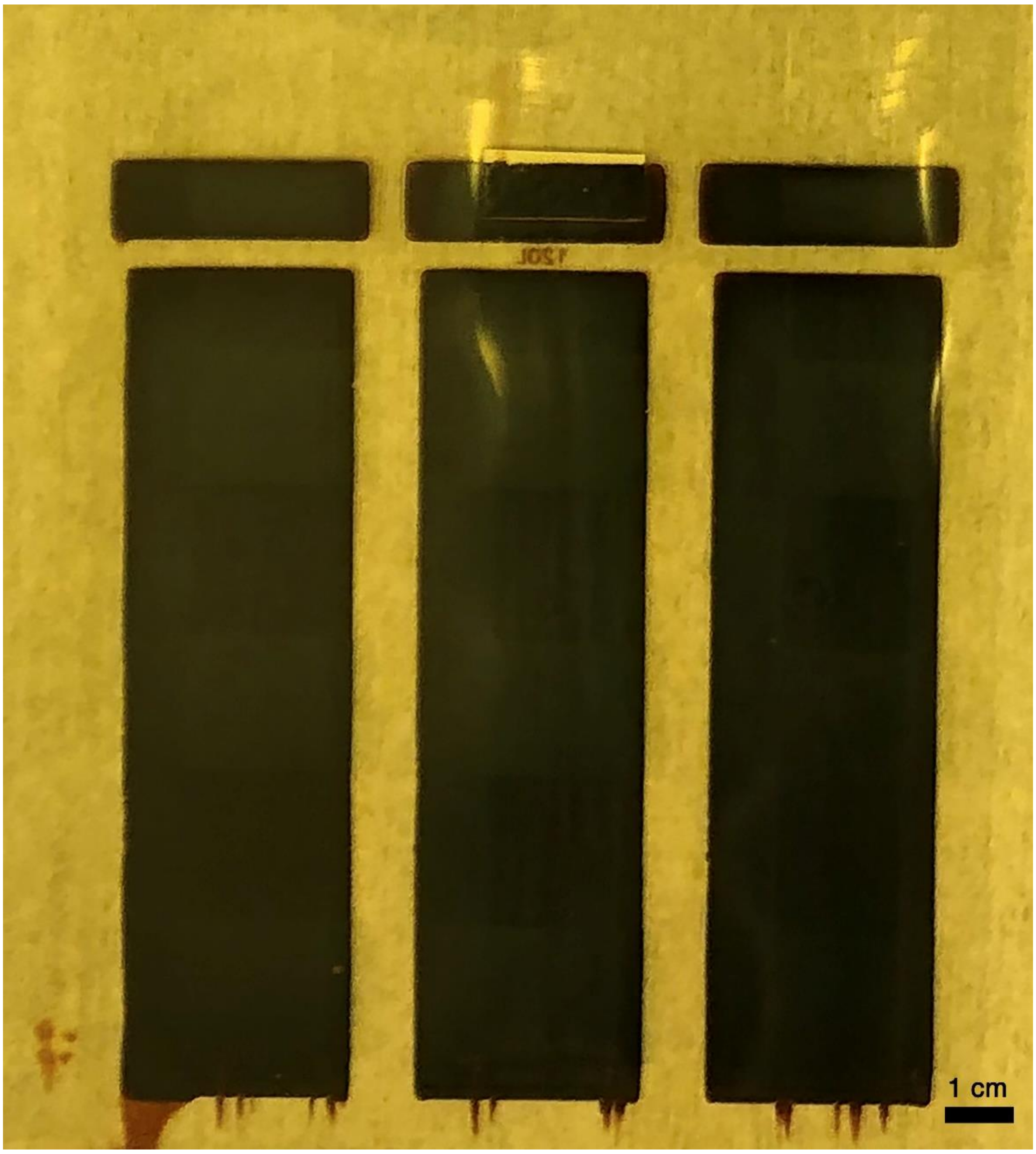

Supplementary Figure 12. A picture of A4-paper-sized film of gravure-printed perovskite on the PET/ITO/SnO_2.

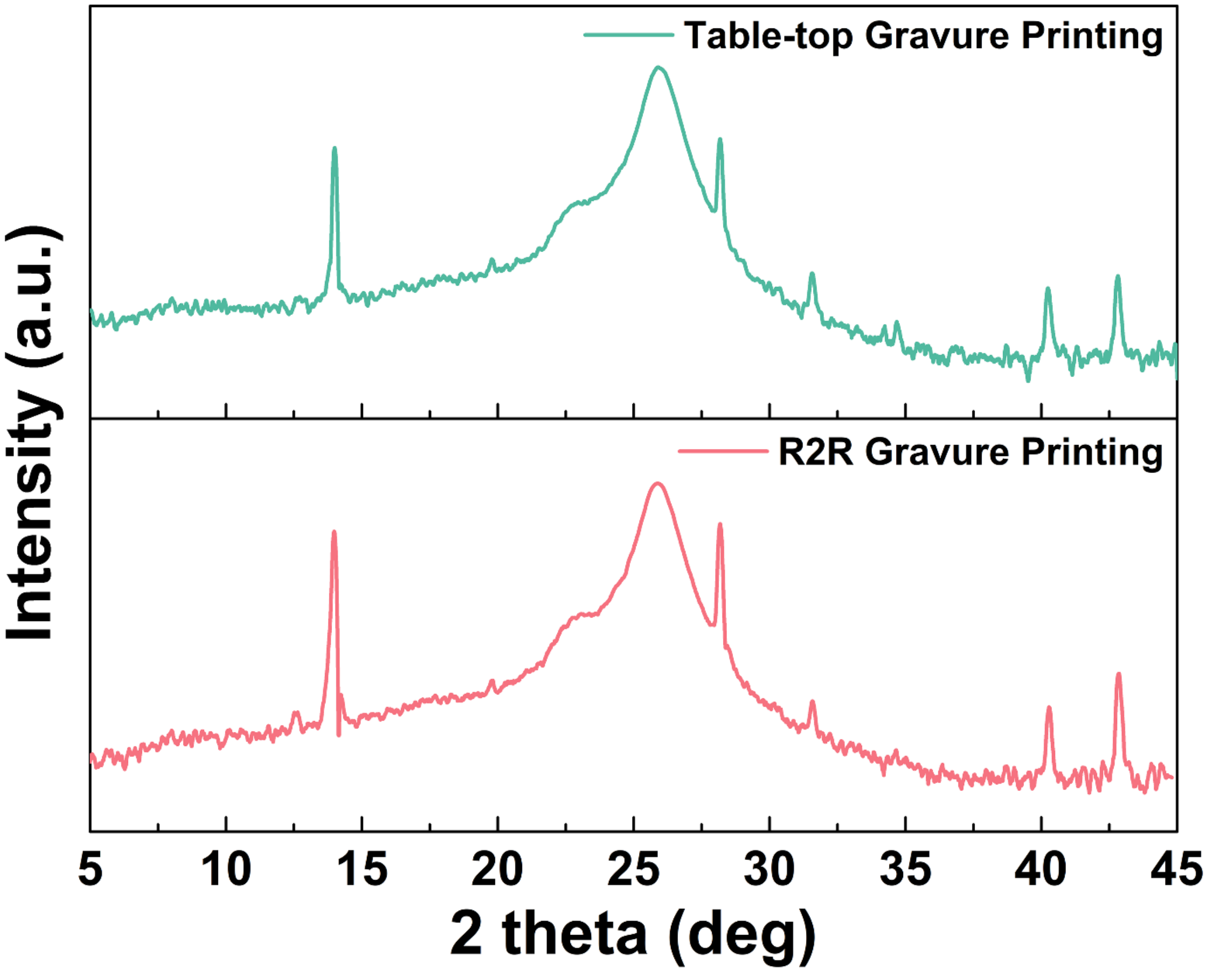

Supplementary Figure 13. XRD spectra of perovskite layer made by table-top or R2R gravure printing.

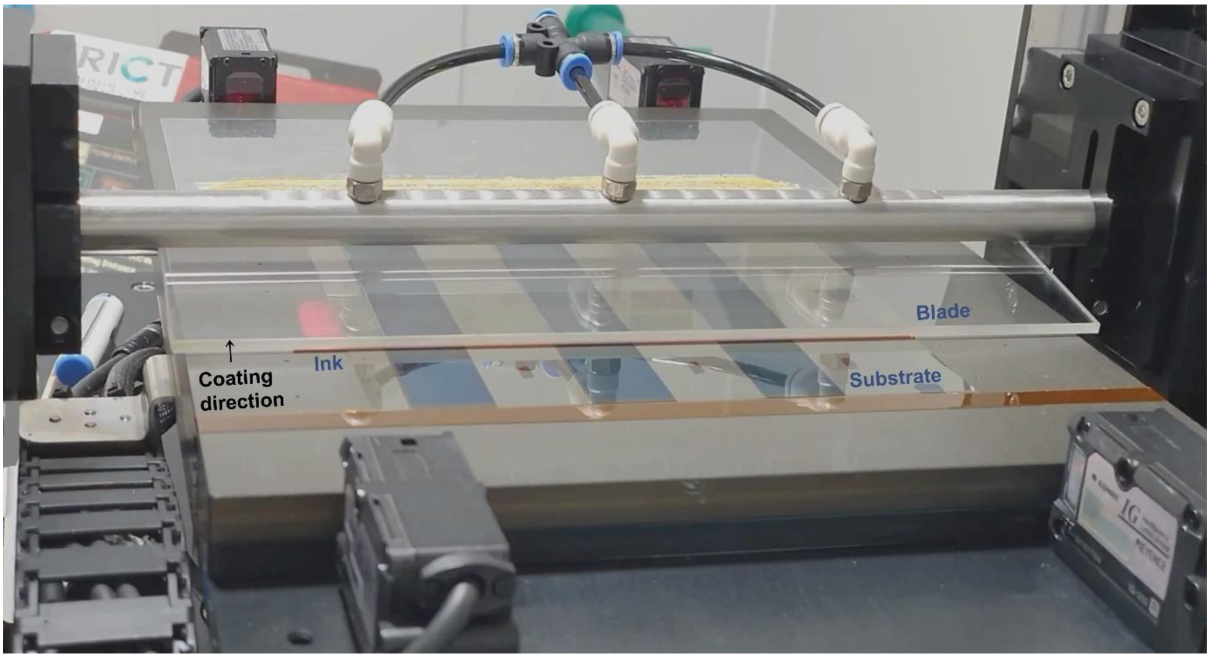

Supplementary Figure 14. A picture illustrating a shear-coating of Spiro-OMeTAD on the top of a perovskite film.

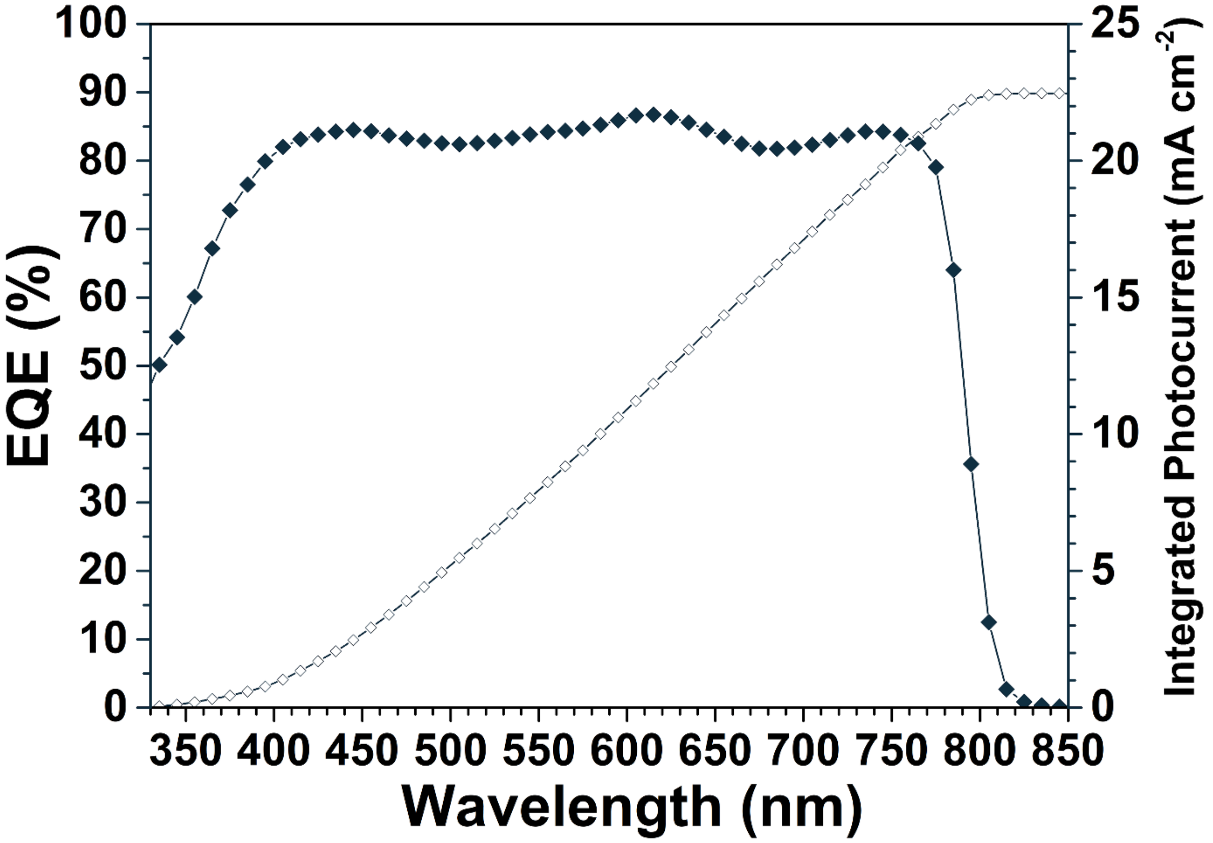

Supplementary Figure 15. EQE spectrum of the PSC with gravure-printed perovskite layer.

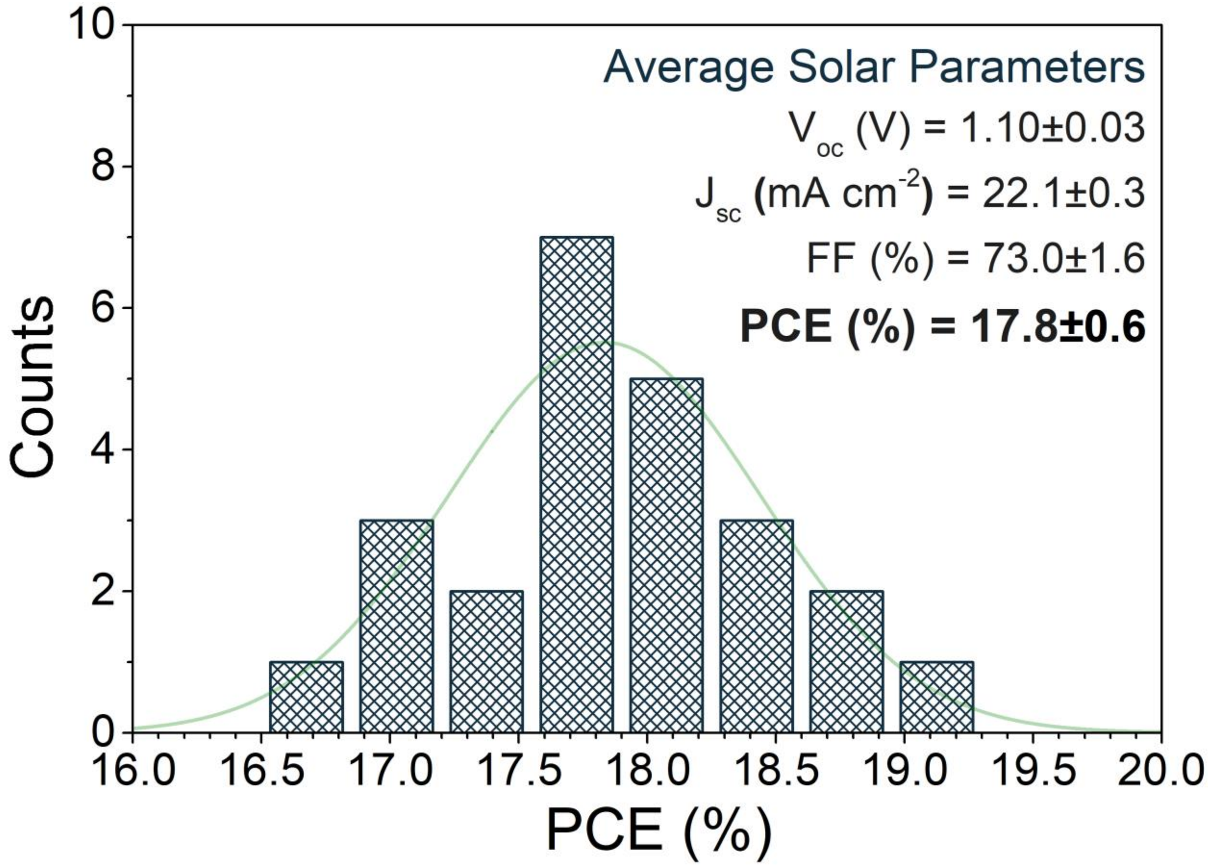

Supplementary Figure 16. PCE distribution of PSCs based on a gravure-printed perovskite layer.

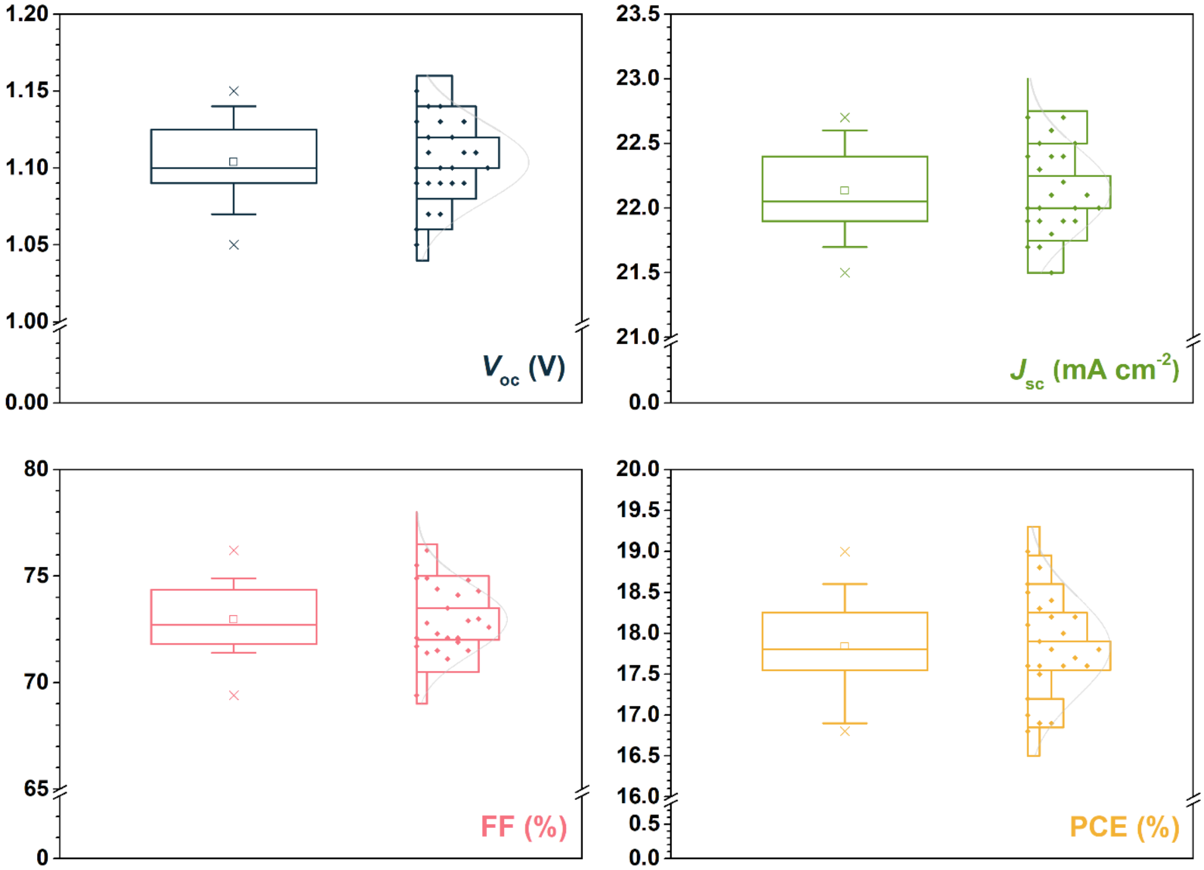

Supplementary Figure 17. Box charts and histograms illustrating the distribution of solar parameters of PSCs based on a gravure-printed perovskite layer. Crossmark in box plot indicates maximum and minimum values, top and bottom bar indicates 5% and 95% values, the upper and lower boundary of the box represents standard deviation from average (± 1.5σ), a square in the middle of the box is median, and the medium line in the box is average value.

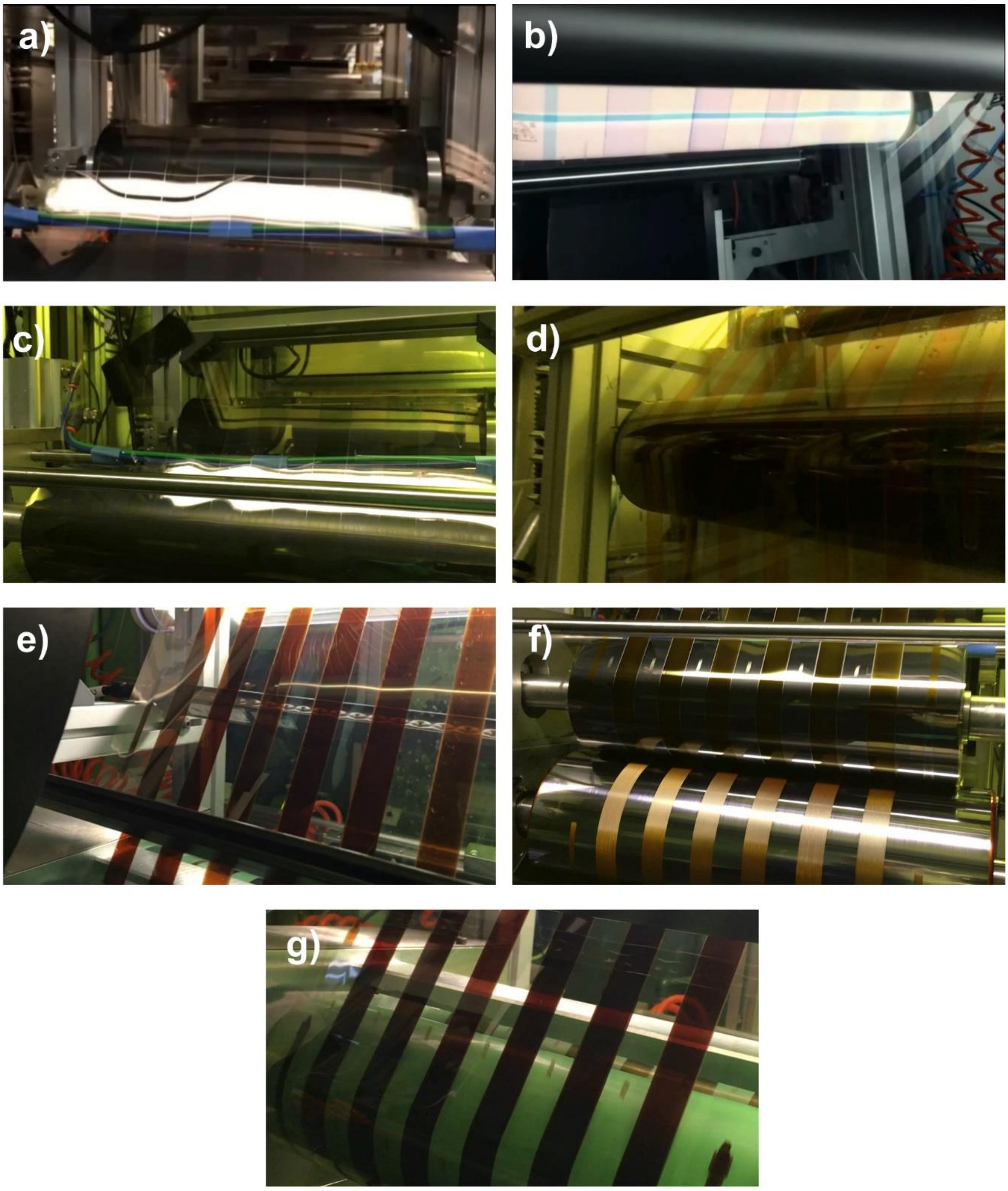

Supplementary Figure 18. R2R processing of consisting layers in PSCs. Each picture represents procedures during subsequent R2R processing. a) Printing of SnO_2 NPs, b) SnO_2 NPs after annealing, c) printing of perovskite precursor, d) perovskite layer right after bathing, e) perovskite layer after annealing, f) printing of P3HT, and g) P3HT layer after annealing.

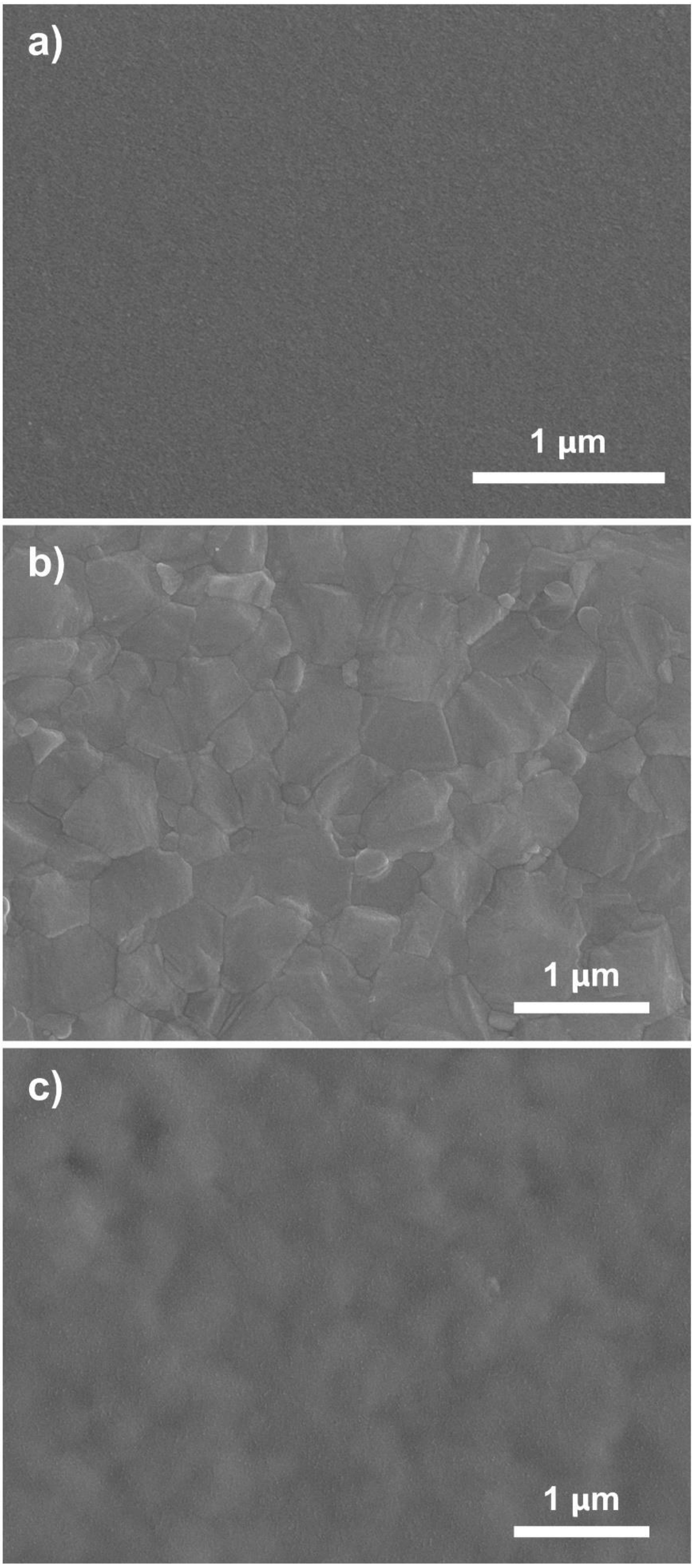

Supplementary Figure 19. SEM images of a) R2R printed SnO_2 NPs, b) R2R printed perovskite, c) R2R printed P3HT layers.

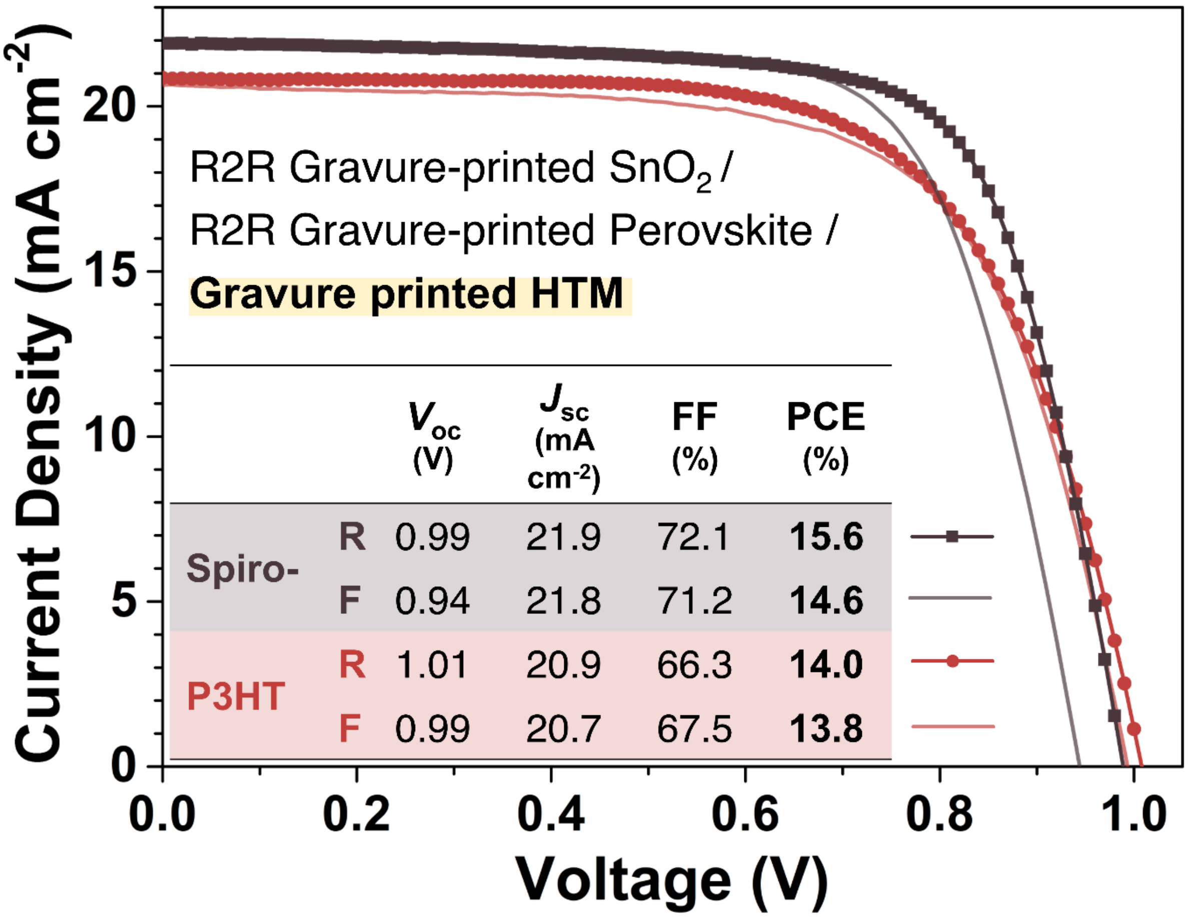

Supplementary Figure 20. Current density-voltage curves of the devices with table-top gravure printed hole-transporting layers on the top of the R2R printed SnO_2/perovskite.

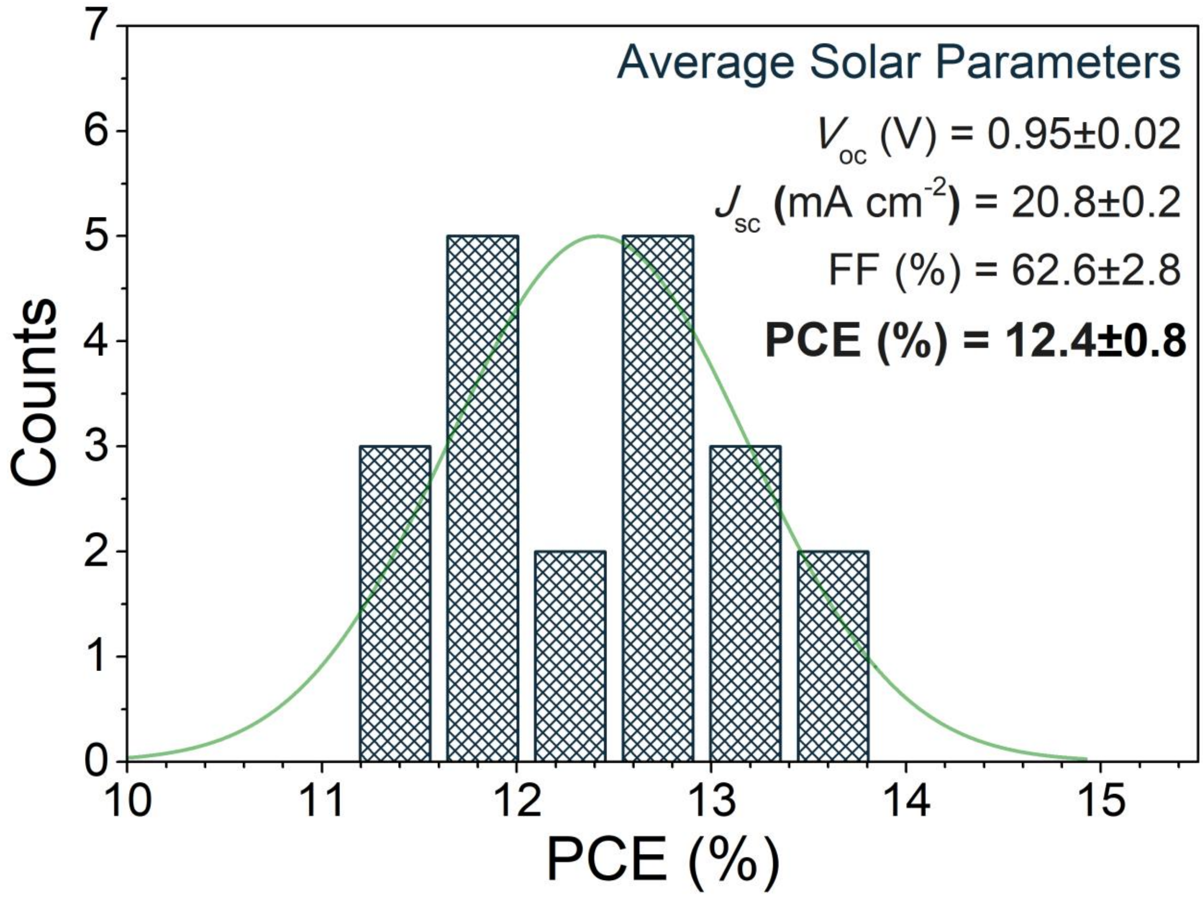

Supplementary Figure 21. PCE distribution of fully R2R processed PSCs.

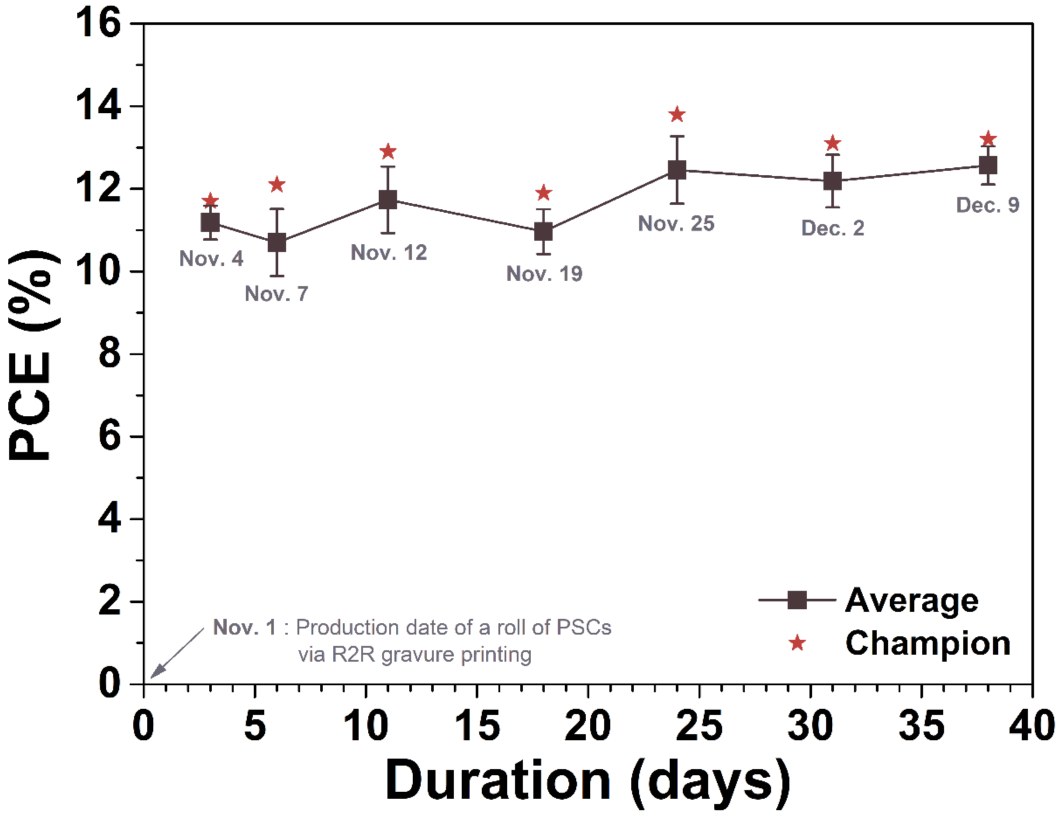

Supplementary Figure 22. Average and champion PCEs of the PSCs, made from different parts of the roll for every few days since the production date of the roll. Average PCE for each day was obtained from at least 12 devices.

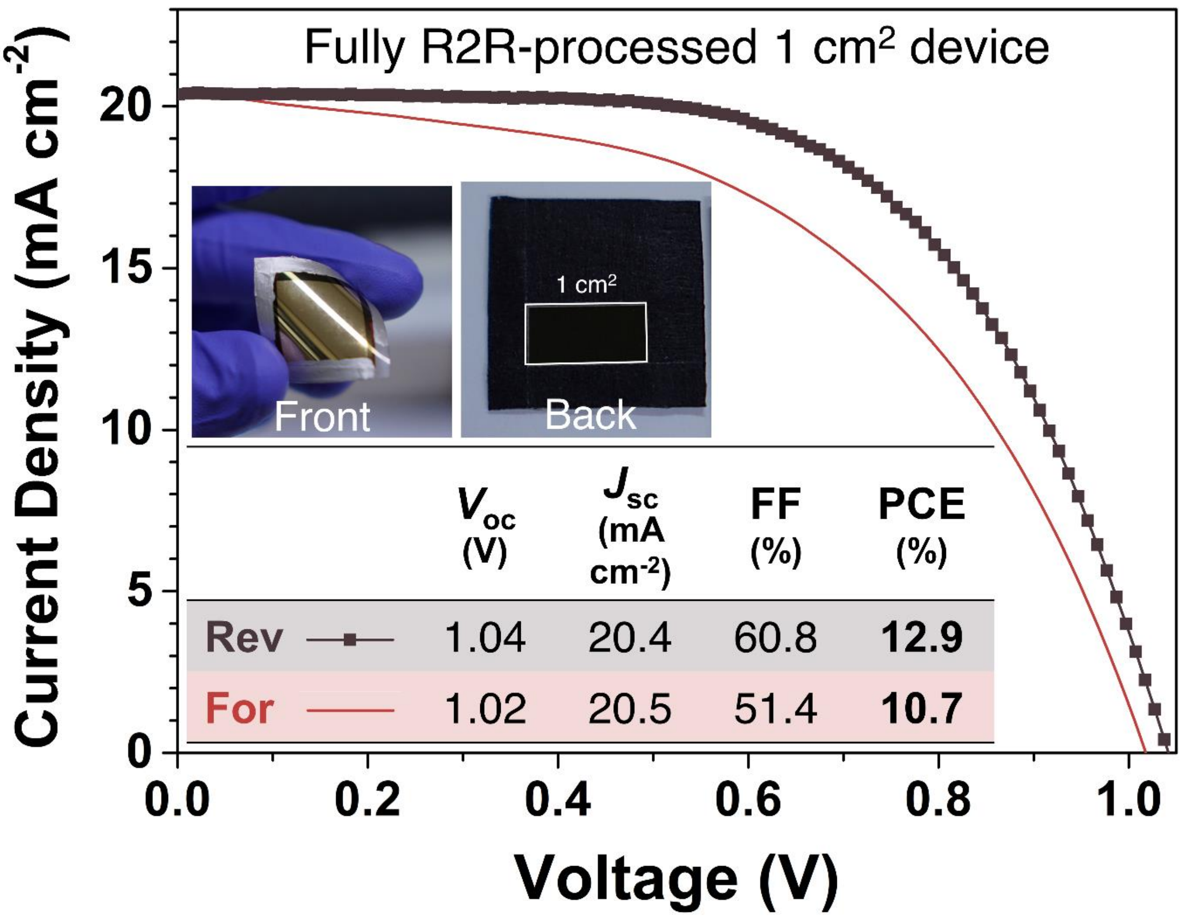

Supplementary Figure 23. Current density – voltage curves of large-area (1 cm^2), fully R2R-processed PSCs measured from both directions.

Supplementary Table 1. The safety issues and basic properties of various antisolvents

	Safety				Properties		
	Severely harmful statements in GHS[a]	Suggestion in previous reports[b]	IDLH[c] [ppm]	LD_{50}[d] [mg/kg]	Boiling point[e] [°C]	Viscosity[f] [mPa·s]	Polarity[g] ($E_T(30)$)
DEE[h]	N	Hazardous	1900	1215	34.6	0.224	34.5
Tol[h]	Y	Problematic	500	>2000	111	0.59	33.9
CB[h]	Y	Problematic	1000	1110	131	0.753	36.8
CF[h]	Y	Hazardous	500	695	61.2	0.563	39.1
Ani[h]	N	Recommended	N/A	3700	154	0.908	37.1
EA[h]	N	Recommended	2000	5620	77.1	0.426	38
nBuOH[h]	N	Recommended	1400	700	117.7	2.6	49.7
IPA[h]	N	Recommended	2000	5045	82.6	1.96	48.4
EtOH[h]	Y	Recommended	3300	6000	78.2	1.1	51.8
tPeOH[h]	N	Recommended	N/A	5184	101	4.47	41.5
tBuOH[h]	N	Recommended	1600	>3100	83	3.39	43.3
tBuOH:EA[i]	-	-	-	-	-	2.77	42.7

[a] GHS: Globally Harmonized System of Classification and Labelling of Chemicals. Severely harmful statements include H340-341 (genetic defects), H350-351 (cancer), H360-361 (damaging fertility or the unborn child), H362 (harm to breast-fed children), and H370-373 (damaging organs).

[b] All the statements are obtained from Ref. 30, except for CB, EA (from Ref. 31), and tPeOH (from Ref. 32)

[c] IDLH: Immediately Dangerous to Life or Health Values. All the values are obtained from the website of the National Institute for Occupational Safety and Health (NIOSH), United States.

[d] LD_{50}: Median Lethal Dose. All the values are obtained from the MSDS documents provided by the supplier, for rat, oral.

[e] Boiling points for various antisolvents are provided from the supplier.

[f] Viscosity values are provided from the supplier.

[g] Ref. 33

[h] DEE: Diethyl ether, Tol: Toluene, CB: Chlorobenzene, CF: Chloroform, Ani: Anisole, EA: Ethyl acetate, nBuOH: n-Butanol, IPA: 2-Propanol, EtOH: Ethanol, tPeOH: tert-Pentanol, tBuOH: tert-Butanol.

[i] The viscosity and polarity values of mixed solvent is estimated by interpolation from the values of pure solvents assuming linear relationship.

Supplementary Table 2. The solar parameters of champion devices produced with various antisolvents.

	V_{oc} [a] [V]	J_{sc} [a] [mA cm^{-2}]	FF[a] [%]	PCE[a] [%]
tBuOH:EA	1.14	23.0	82.6	21.7
Anisole	0.74	21.3	57.8	9.2
DEE	1.07	22.4	75.9	18.2
EA	1.1	22.6	80.3	20
tPeOH	1.13	22.6	82.1	20.9
nBuOH	1.12	22.7	80.5	20.4
IPA	1.12	23.2	76.9	19.9

[a] V_{oc}: Open-circuit voltage, J_{sc}: Short-circuit current density, FF: Fill factor, PCE: Power conversion efficiency.

Supplementary Note 1.

In order to investigate the origin of different behavior of antisolvents during bathing, the antisolvents were directly mixed with PbI_2 or precursor solution. (Supplementary Figure 1) The mixing of PbI_2-N,N-dimethyl formamide (DMF) solution with antisolvents resulted in precipitation of yellow powder (PbI_2), which means all the antisolvents can extract DMF effectively from PbI_2-DMF complex. For PbI_2 in dimethyl sulfoxide (DMSO), DEE is not miscible with DMSO at all. Mixing of IPA and tBuOH:EA precipitated white powder, PbI_2-DMSO complex.[6] EA and Anisole precipitated white powder with yellowish supernatant containing PbI_2. IPA and tBuOH cannot break PbI_2-DMSO bond, but EA and Anisole can extract some of DMSO from PbI_2-DMSO. When the antisolvents were mixed with $(FAPbI_3)_{0.95}(MAPbBr_3)_{0.05}$ solution, DEE showed complete phase separation. EA and Anisole are well-mixed with DMF and DMSO, but they also disperse $(FAPbI_3)_{0.95}(MAPbBr_3)_{0.05}$ in mixed solution, represented by yellowish color of supernatant. Both IPA and tBuOH:EA precipitated $FAPbI_3$ from DMF without make a dispersion. For DMSO solution, tBuOH:EA precipitate mostly yellow crystals (FAI-PbI_2-DMSO complex) but IPA precipitates white powder (PbI_2-DMSO complex), which may originates from the partial dissolution of FAI in IPA. It is elucidated from this result that tBuOH:EA most effectively work as an antisolvent by extracting DMF and excess DMSO solvent from solution while not dissolving FAI from the precursor.
