# Supplementary material for: Roll-to-roll gravure-printed flexible perovskite solar cells using eco-friendly antisolvent bathing with wide processing window
Source: Nat Commun. 2020 Oct 13;11:5146. doi: 10.1038/s41467-020-18940-5 (PMC7555830; doi:10.1038/s41467-020-18940-5)
Supplement: Supplementary file 2 — Description of Additional Supplementary Files [file 41467_2020_18940_MOESM2_ESM.pdf]

Description of Additional Supplementary Files

File Name: Supplementary Movie 1
Description: R2R Gravure-printing of flexible PSCs
