# Supplementary material for: Roll-to-roll gravure-printed flexible perovskite solar cells using eco-friendly antisolvent bathing with wide processing window
Source: Nat Commun. 2020 Oct 13;11:5146. doi: 10.1038/s41467-020-18940-5 (PMC7555830; doi:10.1038/s41467-020-18940-5)
Supplement: Supplementary file 4 — Reporting Summary [file 41467_2020_18940_MOESM4_ESM.pdf]

nature research

Corresponding author(s): Jangwon Seo

Solar Cells Reporting Summary

Nature Research wishes to improve the reproducibility of the work that we publish. This form is intended for publication with all accepted papers reporting the characterization of photovoltaic devices and provides structure for consistency and transparency in reporting. Some list items might not apply to an individual manuscript, but all fields must be completed for clarity.

For further information on Nature Research policies, including our data availability policy, see Authors & Referees.

Experimental design

Please check: are the following details reported in the manuscript?

1. Dimensions

Item	Yes	No	Details
Area of the tested solar cells	☒	☐	Methods Section; 0.096 cm2
Method used to determine the device area	☒	☐	Methods Section; measured by optical microscope

2. Current-voltage characterization

Item	Yes	No	Details
Current density-voltage (J-V) plots in both forward and backward direction	☒	☐	Figure 3d, Figure 4e, Figure 6f
Voltage scan conditions<br>*For instance: scan direction, speed, dwell times*	☒	☐	Methods Section; Reverse (1.2 to -0.2V), Forward (-0.2 to 1.2V), delay time of 10 ms.
Test environment<br>*For instance: characterization temperature, in air or in glove box*	☒	☐	Ambient environment (25 °C, 30-40% RH)
Protocol for preconditioning of the device before its characterization	☐	☒	No preconditioning step was used.
Stability of the J-V characteristic<br>*Verified with time evolution of the maximum power point or with the photocurrent at maximum power point; see ref. 7 for details.*	☒	☐	Figure 4f; Maximum powerpoint tracking measurement

3. Hysteresis or any other unusual behaviour

Item	Yes	No	Details
Description of the unusual behaviour observed during the characterization	☒	☐	No unusual behavior was observed during measurement. A small amount of hysteresis was observed.
Related experimental data	☒	☐	Figure 3d, Figure 4e, Figure 6f

4. Efficiency

Item	Yes	No	Details
External quantum efficiency (EQE) or incident photons to current efficiency (IPCE)	☒	☐	Supplementary Figure 15
A comparison between the integrated response under the standard reference spectrum and the response measure under the simulator	☒	☐	Short-circuit current density was well-matched with the value obtained from external quantum efficiency measurement. (Figure 4f and Supplementary Figure 15)
For tandem solar cells, the bias illumination and bias voltage used for each subcell	☐	☒	No tandem cells were fabricated in this manuscript.

5. Calibration

Item	Yes	No	Details
Light source and reference cell or sensor used for the characterization	☒	☐	Methods Section; Solar simulator (Newport, Oriel Class A, 91195A)
Confirmation that the reference cell was calibrated and certified	☒	☐	Methods Section; Light intensity was calibrated Si-reference cell certified by the NREL, USA.

Calculation of spectral mismatch between the reference cell and the devices under test

- [x] Yes
- [ ] No

It has been calibrated.

6. Mask/aperture

Size of the mask/aperture used during testing

- [x] Yes
- [ ] No

Methods Section; 0.096 cm2 of mask was used for testing.

Variation of the measured short-circuit current density with the mask/aperture area

- [ ] Yes
- [x] No

We don't added the data; but we observed that there is no obvious changes in short-circuit current upon varying the size of active area.

7. Performance certification

Identity of the independent certification laboratory that confirmed the photovoltaic performance

- [ ] Yes
- [x] No

It was not certified.

A copy of any certificate(s)
Provide in Supplementary Information

- [ ] Yes
- [x] No

It was not certified.

8. Statistics

Number of solar cells tested

- [x] Yes
- [ ] No

At least 24 devices each for glass-based, table-top gravure-printed, and R2R gravure-printed devices were tested.

Statistical analysis of the device performance

- [x] Yes
- [ ] No

Figure 3a-c, Supplementary Figure 16-17, 21.

9. Long-term stability analysis

Type of analysis, bias conditions and environmental conditions
For instance: illumination type, temperature, atmosphere humidity, encapsulation method, preconditioning temperature

- [x] Yes
- [ ] No

Section "Pilot-scale R2R gravure-printing of flexible PSCs" and Supplementary Figure 22.
